# Temporal regulation of the Mediator complex during muscle proliferation, differentiation, regeneration, aging, and disease

**DOI:** 10.3389/fcell.2024.1331563

**Published:** 2024-04-16

**Authors:** Dominic W. Kolonay, Kristina M. Sattler, Corinne Strawser, Jill Rafael-Fortney, Maria M. Mihaylova, Katherine E. Miller, Christoph Lepper, Kedryn K. Baskin

**Affiliations:** ^1^ Department of Physiology and Cell Biology, The Ohio State University Wexner Medical Center, Columbus, OH, United States; ^2^ Dorothy M. Davis Heart and Lung Research Institute, The Ohio State University Wexner Medical Center, Columbus, OH, United States; ^3^ Department of Pediatrics, The Ohio State University Wexner Medical Center, Columbus, OH, United States; ^4^ Institute for Genomic Medicine, Nationwide Children’s Hospital, Columbus, OH, United States; ^5^ Department of Biological Chemistry and Pharmacology, The Ohio State University Wexner Medical Center, Columbus, OH, United States; ^6^ The Ohio State University Comprehensive Cancer Center, The Ohio State University, Columbus, OH, United States

**Keywords:** Mediator complex, transcription, myogenesis, cell differentiation, skeletal muscle regeneration

## Abstract

Genesis of skeletal muscle relies on the differentiation and fusion of mono-nucleated muscle progenitor cells into the multi-nucleated muscle fiber syncytium. The temporally-controlled cellular and morphogenetic changes underlying this process are initiated by a series of highly coordinated transcription programs. At the core, the myogenic differentiation cascade is driven by muscle-specific transcription factors, i.e., the Myogenic Regulatory Factors (MRFs). Despite extensive knowledge on the function of individual MRFs, very little is known about how they are coordinated. Ultimately, highly specific coordination of these transcription programs is critical for their masterfully timed transitions, which in turn facilitates the intricate generation of skeletal muscle fibers from a naïve pool of progenitor cells. The Mediator complex links basal transcriptional machinery and transcription factors to regulate transcription and could be the integral component that coordinates transcription factor function during muscle differentiation, growth, and maturation. In this study, we systematically deciphered the changes in Mediator complex subunit expression in skeletal muscle development, regeneration, aging, and disease. We incorporated our *in vitro* and *in vivo* experimental results with analysis of publicly available RNA-seq and single nuclei RNA-seq datasets and uncovered the regulation of Mediator subunits in different physiological and temporal contexts. Our experimental results revealed that Mediator subunit expression during myogenesis is highly dynamic. We also discovered unique temporal patterns of Mediator expression in muscle stem cells after injury and during the early regeneration period, suggesting that Mediator subunits may have unique contributions to directing muscle stem cell fate. Although we observed few changes in Mediator subunit expression in aging muscles compared to younger muscles, we uncovered extensive heterogeneity of Mediator subunit expression in dystrophic muscle nuclei, characteristic of chronic muscle degeneration and regeneration cycles. Taken together, our study provides a glimpse of the complex regulation of Mediator subunit expression in the skeletal muscle cell lineage and serves as a springboard for mechanistic studies into the function of individual Mediator subunits in skeletal muscle.

## 1 Introduction

The Mediator complex is a general regulator of transcription. It serves as a bridge to facilitate functional interactions between the basal transcriptional machinery, general transcription factors and cofactors to orchestrate transcription through RNA Polymerase II (El Khattabi et al., 2019; Richter et al., 2022). In mammals, Mediator is a multiprotein complex comprised of 26–30 unique proteins that are ubiquitously expressed, and are organized into the Tail, Middle, Head, and Kinase submodules (Flanagan et al., 1991; Thompson et al., 1993; Kim et al., 1994; Fondell et al., 1996). Structural studies have provided insight into the organization of the mammalian Mediator complex; however, little is known regarding the function of individual Mediator subunits (Chen et al., 2021; Rengachari et al., 2021). Constitutive knockout mouse models of several Mediator genes result in embryonic lethality, shedding light onto the importance of subunits during embryonic development (Ito et al., 2000; Westerling et al., 2007; Rocha et al., 2010; Miao et al., 2018). By contrast, only a few recent studies using conditional knockout mouse models have begun to reveal the cell-specific and developmental-specific functions of Mediator genes (Chen et al., 2010; Grueter et al., 2012; Baskin et al., 2014; Amoasii et al., 2016; Baskin et al., 2017).

There is increasing evidence that Mediator is required for cell fate determination. For example, in embryonic stem cells, master transcription factors recruit Mediator to enhancer regions that activate pluripotent gene expression programs (Whyte et al., 2013). Mediator is critical for hematopoietic stem cell homeostasis by co-localizing with hematopoietic transcription factors to maintain expression of hematopoietic genes (Aranda-Orgilles et al., 2016). In neural stem cells, Mediator interacts with transcription factors and localizes to enhancers that are critical for transcription of genes driving neurogenesis (Quevedo et al., 2019). However, the requirement of Mediator for muscle stem cell fate and the role of Mediator in skeletal muscle development have not been investigated.

Myogenic differentiation progresses through temporally well-defined stages: from naive progenitor, to committed myoblast, to differentiated, post-mitotic myocyte and ultimately, to the muscle fiber syncytium via myocyte fusion (Hawke and Garry, 2001). Large, yet very specific changes in the transcriptional program underlie the progression from one stage to the next. Myogenic regulatory factors (MRFs), basic helix-loop-helix transcription factors including MYF5, MYOD, MYOG/Myogenin, and MYF6/MRF4 are critical for muscle differentiation (Chal and Pourquie, 2017; Hernandez-Hernandez et al., 2017; Zammit, 2017). However, a thorough understanding of how gene expression programs are collectively regulated in a coordinated manner in skeletal muscle development and regeneration is lacking.

Here we set out to gain a comprehensive understanding of gene regulatory changes of individual components of the Mediator complex during muscle proliferation, differentiation, regeneration, aging, and disease. Using an integrative approach, we combined our experimental findings with our analysis of published sequencing datasets and discovered the temporal, functional, and disease implications of Mediator in skeletal muscle. We found that expression of most Mediator subunits is much higher in activated muscle stem cells and proliferating myoblasts compared to their differentiated multinucleated progeny, the myofiber. Interestingly, we observed very few changes in Mediator with muscle aging, but in diseased muscle, Mediator subunits were significantly dysregulated. Taken together, our study systematically uncovered Mediator subunit expression patterns throughout the life cycle of muscle. Based on our findings, we propose that the Mediator complex coordinates transcriptional regulation of skeletal muscle development and plays an important role in regulating muscle stem cell fate.

## 2 Materials and methods

### 2.1 Mouse studies

Wild type and *mdx* (C57BL/10) male and female mice were bred in-house in a room-temperature and light controlled room with a 12/12 h light/dark cycle, and mice aged 10 weeks were used for experiments. Food and water were provided *ad libitum*. Timed mouse matings were performed using wildtype (C57BL/6J) male and female mice to obtain developmental muscle samples, and 3- and 24-months-old wildtype (C57BL/6J) male mice were used for aging studies. The animal studies were reviewed and approved by The Ohio State University Institutional Animal Care and Use Committee.

### 2.2 Barium chloride-induced acute muscle injury

Wild type (C57BL/10) male and female mice 8–10 weeks-of-age were anesthetized with isoflurane, and hair on the distal portion of both lower legs was removed with Nair Lotion (Church and Dwight Co., Ewing, NJ). The leg was rinsed with sterile water and dried. The mice were injected (Becton Dickinson, Franklin Lakes, NJ, 3/10 cc U-100 Insulin syringe, 30G × 3/8″needle) intramuscularly into the middle portion of the mouse’s left tibialis anterior (TA) muscle with 50 μL of sterile 1.2% barium chloride (Sigma-Aldrich, St. Louis, MO, B0750) diluted in sterile water as previously described (Hauck et al., 2019). To serve as a control, the right TA muscle was injected with 50 μL of sterile saline. Animals were put into a warm chamber for recovery, prior to being transferred back into regular housing cages with food at the cage bed. Their health was monitored each day post injury. No animal in this study met early removal criteria. Mice were euthanized, and TA muscles were harvested 4 days post-injury. All procedures were approved by The Ohio State University’s Institutional Animal Care and Use Committee.

### 2.3 Isolation and culture of mouse primary myoblasts

Primary myoblasts were isolated from wild type (C57BL/6J) mice via Magnetic Activated Cell Sorting (MACS). Hindlimb muscles were minced and enzymatically digested with 750 U/mL Collagenase Type II (Worthington) in Wash Medium (WM, Ham’s F-10, 10% horse serum, 1% Penicillin-Streptomycin (Pen/Strep, Gibco) in a shaking water bath at 37°C for 90 min. Tissue slurries were diluted 10-fold with WM and centrifuged at 500 *g* at 4°C for 10 min. Pelleted cells were resuspended in fresh WM and digested with 100 U/mL Collagenase Type II and 1.1 U/mL Dispase (ThermoFisher Scientific) in a shaking water bath at 37°C for 30 min. Samples were drawn and expelled ten times with a syringe and 20G needle, then filtered through a 70 μm cell strainer, centrifuged, and resuspended in 2 mL MACS buffer (0.5% Bovine Serum Albumin (Sigma), 2 mM EDTA in 1X Phosphate-Buffered Saline, filter-sterilized). Slurries were filtered through a 40 μm filter, centrifuged, and resuspended in 160 μL MACS buffer before proceeding to the MACS protocol.

MACS isolation was performed following instructions provided by the MACS Satellite Cell Isolation Kit (Miltenyi Biotec) and duplicating the negative cell selection step. In brief, 40 μL SC isolation kit beads were added to the 160 μL muscle tissue slurry and incubated on ice with shaking for 15 min. Cell slurries were applied to freshly prepared MACS LS columns on a magnetic stand and washed with 2 mL MACS buffer. The flow-through was collected and centrifuged at 1,000 x g at 4°C for 5 min. Cell pellets were resuspended in 80 μL MACS buffer and 20 μL SC isolation kit beads were added for a second round of negative selection. For positive selection, cell pellets were resuspended in 80 μL MACS buffer, to which 20 μL Anti-Integrin α-7 Microbeads (Miltenyi Biotec) were added and incubated on ice with shaking for 15 min. Cell slurries were applied to freshly prepared MACS MS columns and washed with 1 mL MACS buffer. MS columns were removed from MACS separator magnet and attached to collection tubes. Primary myoblasts were eluted with 1.5 mL MACS buffer and subsequently centrifuged. Cell pellets were re-suspended in 2 mL Myoblast Growth Medium (20% ES-cell grade Fetal Bovine Serum, 10% Horse Serum, 0.5% Chick Embryo Extract, 1% Pen/Strep in Ham’s F-10, 2 ng/mL bFGF) and plated on Matrigel-coated (ThermoFisher Scientific) tissue culture dishes. Myoblasts were differentiated at 85%–90% confluency in DMEM supplemented with 2% horse serum and 1% Pen/Strep.

### 2.4 Cell culture

C2C12 myoblast cells (ATCC) were cultured in Dulbecco’s modified Eagle medium (DMEM, 1.5 g/L glucose, Corning) with 10% fetal bovine serum and 1% Pen/Strep for growth and maintenance. Myoblasts were differentiated into myotubes using DMEM with 2% horse serum and 1% Pen/Strep.

### 2.5 Immunofluorescence

Mouse primary myoblasts were plated on two 8-chamber slides (Cat: 177445, ThermoFisher Scientific) and cultured overnight in Myoblast Growth Medium. One slide was fixed in 2% PFA for 10 minutes at room temperature, while the second slide was washed with 1X PBS and switched to Differentiation Medium (DMEM supplemented with 2% Fetal Bovine Serum and 1X Pen-Strep) and cultured for 4 days with daily media changes before fixing with 2% PFA for 10 minutes at room temperature.

Fixed samples were permeabilized in 0.3% triton-X-100 for 5 minutes, washed with PBT (0.05% triton-x in PBS), then incubated in goat blocking solution (10% heat-inactivated goat serum in PBT) for 1 hour at room temperature. Samples were incubated with anti-Desmin polyclonal antibody (1:200 in goat block, Cat: PA5-16705, ThermoFisher Scientific) for 2 h at room temperature, washed with PBT (3 washes, 5 min each), then incubated with anti-Rabbit IgG Alexa-fluor 568 secondary antibody (1:1,000 in goat block, Cat: A-11011, ThermoFisher Scientific) for 90 min at room temperature protected from light. Samples were then counterstained with 4′,6-diamidino-2-phenylindole (DAPI) at 1 μg/mL in PBT for 10 min, washed with PBT (2 washes, 5 min each), and Fluoromount-G mounting medium (Cat: 00–4,958–02, ThermoFisher Scientific) was used to mount coverslips. Slides were imaged using a Zeiss Axioskop microscope with a Zeiss AxioCam monochrome charge-coupled device (CCD) camera. Images were merged and pseudo-colored in ImageJ.

### 2.6 Sample collection and preparation

For RNA isolation, myoblasts and myotubes were collected in 500 μL Tri-Reagent (Sigma-Aldrich) and stored at −20°C until processing. For protein isolation, cells were collected in 200 μL of RIPA buffer (Millipore) containing PhosStop phosphatase inhibitor cocktail tablets (Roche) and cOmplete Mini, EDTA-free protease inhibitor cocktail tablets (Roche) on ice. Samples were then incubated on ice for 30 min with vortexing to ensure lysis and then centrifuged for 10 min at 16,000 x g at 4°C. Supernatants were collected and stored at −20°C.

Mice were euthanized and muscles were dissected and snap-frozen in liquid nitrogen and stored at −80°C. Tissue was lyophilized for 8 h and pulverized with ceramic beads on dry ice using a Bertin Technologies Percellys Evolution Tissue Homogenizer (Molnar et al., 2021). Pulverized tissues were then homogenized on ice with a Polytron PT1200-E in either 500 μL Tri-Reagent or 100 μL RIPA buffer supplemented with protease and phosphatase inhibitor cocktail tablets (Roche). Homogenized tissues in Tri-Reagent were stored at −20°C. Homogenized tissues in RIPA buffer were centrifuged for 10 min at 16,000 x g at 4°C, and the supernatant was stored at −20°C.

### 2.7 RNA isolation and reverse transcription quantitative polymerase chain reaction (RT-qPCR)

Total RNA was extracted from cells or dissected tissues using the Tri-Reagent protocol (Sigma Aldrich). RNA purity and concentration were assessed via NanoDrop One Spectrophotometer (ThermoFisher Scientific). cDNA was reverse transcribed from 1 μg of total RNA using the iScript cDNA Synthesis kit (BioRad) according to the manufacturer’s protocol. qPCR reactions were run using iTaq SYBR Green Universal Supermix (BioRad) with 20 ng of cDNA in a CFX384 Real Time System (BioRad) using validated and optimized target-specific primers. Relative gene expression levels were calculated using the DDCT method normalized to *18S* and expressed as fold change or log_2_ (fold change) relative to the control condition.

The following primers were used for qPCR:

**Table:** 

Primer name	Forward primer sequence	Reverse primer sequence
*Med1*	GAA​TGG​ACT​GGG​CTC​TCA​CC	AGC​TCA​CAG​GAT​TCT​CCC​CA
*Med4*	CCG​AGC​AGA​TCC​TGG​CAA​C	TGG​AGC​ACA​GAC​AGC​ATT​GC
*Med6*	CCG​AGA​TAA​CCT​GCT​GGG​G	GCC​TCT​GCA​TTT​TGA​CCA​CC
*Med7*	GGC​TGC​TCT​GGT​CTT​AGG​TTC	GGA​GCC​AAG​CCT​TCC​TGA​A
*Med8*	CCG​GCC​AAA​CAA​GCA​GAC​T	CCC​TGC​AAG​GAT​TGT​ACC​AGC
*Med9*	GCA​TGG​ACA​AGG​ACA​GCC​C	GTC​CTC​ACT​TGC​TCT​CGG​AGG
*Med10*	GGG​GCT​GAG​CCA​GAA​GCT​AA	GTG​TAG​AGC​TGG​GGA​TTC​CGA​C
*Med11*	ATG​GCT​ACC​TAC​AGT​CTG​GC	CAG​TTC​CAG​GAT​CGC​CGT​TC
*Med12*	GGT​TCT​ACT​ATA​GGG​CCT​TTG​C	TCC​AGC​ATT​CCA​TCC​TGA​AAC
*Med13*	CCT​ATG​AAT​GCC​GTA​CTT​TGC​T	ACC​ACT​TGC​CAA​TTC​GTA​CAA
*Med14*	GCAAGGACCATCCCGACA	ACA​CAC​ACT​CGT​ATA​AAC​GGG​C
*Med15*	GTC​AGC​CAA​ATT​GAG​GAT​GCC	GCC​TTC​AGG​AAC​ACA​TGA​CTC
*Med16*	CCT​ACG​CAA​TGA​TGA​CCA​GGA​T	AGC​ATC​AGC​AGA​CAG​TAG​CC
*Med17*	ATG​GCA​CCG​AGA​CGT​ACC​T	GGG​CAC​TTC​GCA​AAT​TGT​TCC
*Med18*	CAC​TGG​GGG​CAC​CAT​TAA​CAT	CTT​AGG​ACG​AAC​GGG​CTG​G
*Med19*	CAT​GCT​GTC​TGG​TTT​CCG​C	GGC​TCT​GTT​TGT​GCT​TAT​GCT​TA
*Med20*	AGG​TGG​AGT​ATG​GCC​CTT​GT	GCA​TCG​TGT​CTG​TTC​CCA​AAT
*Med21*	CGCTGTGAACTCGCTTGC	ACT​CTT​CTG​TAG​GAT​TGG​CTG​G
*Med22*	GCG​GCT​CAA​AGA​CGA​CAT​CA	CGT​GCA​TCT​CAT​AAT​TGT​CCT​GT
*Med23*	CCA​TTT​GTG​GCC​GAT​GCA​G	CAA​CAA​AGC​AGT​CTG​CGG​TTC
*Med24*	ACG​CCA​TTA​GTT​CCC​AGA​TGG	CGTGGCAACTGAGTCGGT
*Med25*	GGA​GAC​TAT​GGT​GGA​ACC​CAG	CAT​GAA​CTT​GAT​GCC​ATC​GAG​C
*Med26*	CGA​CTC​CCA​GAG​CAA​CAT​CC	TGA​GCT​TCC​CTA​GTC​GTG​TCT
*Med27*	CCA​CCT​CAG​TAT​GTC​GAT​GAC​G	CCT​TCC​CCA​AGG​TCA​CCA​G
*Med28*	GCC​CCG​AGA​CCA​TCT​AAC​AG	CTG​GAC​AGA​TAA​CTG​CAA​CCT​T
*Med29*	CCA​GAC​TTT​GAT​GAA​GGT​TGC​AGC	GCG​CAA​GCA​GAG​TTC​CAG​C
*Med30*	ACC​AAG​ACC​GGC​TAA​CAA​AGC	CAG​TTG​CTC​AAC​AGG​AAT​GGG​G
*Med31*	TGT​TTA​GCC​AAC​CCA​AAC​TAC​C	TCA​TAC​TGG​AGC​AGC​TCT​AAC​AT
*Cdk8*	GCA​CAG​GGA​TTT​GAA​ACC​TGC	GCA​AAG​CCC​ATG​TCA​GCA​AT
*CcnC*	CCC​CTT​GCA​TGG​AGG​ATA​GTG	CTT​TCT​GTT​GTA​CGA​CAC​AGG​C
*Myod1*	CCA​CTC​CGG​GAC​ATA​GAC​TTG	AAA​AGC​GCA​GGT​CTG​GTG​AG
*Myog*	GAG​ACA​TCC​CCC​TAT​TTC​TAC​CA	GCT​CAG​TCC​GCT​CAT​AGC​C
*Tmem8c*	ATC​GCT​ACC​AAG​AGG​CGT​T	CAC​AGC​ACA​GAC​AAA​CCA​GG
*Myh1*	GCG​AAT​CGA​GGC​TCA​GAA​CAA	GTA​GTT​CCG​CCT​TCG​GTC​TTG
*Myh2*	ACT​TTG​GCA​CTA​CGG​GGA​AAC	CAG​CAG​CAT​TTC​GAT​CAG​CTC
*Myh3*	CCA​AAA​CCT​ACT​GCT​TTG​TGG​T	GGG​TGG​GTT​CAT​GGC​ATA​CA
*Myh4*	CCG​CAT​CTG​CAG​GAA​GGG​G	GTG​ACC​GAA​TTT​GTA​CTG​AGT​GT
*Myh8*	GGA​GAG​GAT​TGA​GGC​CCA​AAA	CAC​GGT​CAC​TTT​CCC​TCC​ATC

### 2.8 Western blot analysis

Protein supernatants from C2C12 cells or tissues in RIPA were thawed on ice and protein concentration was determined using the Bradford protein assay according to manufacturer’s protocol (BioRad). Protein supernatants from primary myoblasts in RIPA were thawed on ice and protein concentration was determined using the BCA Protein Assay according to manufacturer’s protocol (Pierce). AllBlue protein ladder (BioRad) was used as a molecular weight marker, and equal amounts of protein from all samples (between 2 and 10 μg depending on which protein was to be detected) was loaded per well into either pre-cast 4%–20% Stain Free gradient gels (BioRad) (C2C12 myoblasts and myotubes, injured mouse muscle, young and aging mouse muscle) or self-cast gels ranging from 7.5%–15% (mouse primary myoblasts and myotubes). Gels were run at 125 V for 50 min in running buffer (Tris/Glycine/SDS Buffer; BioRad). Stain Free images were taken of gels to ensure even protein loading using a ChemiDoc MP Imaging system (BioRad). Wet transfers were performed using a methanol-activated PVDF membranes (Milipore Immobilon) or nitrocellulose membranes (ThermoFisher Scientific) at 90V for 90 min in (Tris/Glycine Buffer; BioRad). Stain Free images of activated membranes were taken to verify transfer. Membranes were also stained with Ponceau S solution (ThermoFisher Scientific) for 15 min, then rinsed with Milli-Q^®^ water to visualize equal sample loading and membrane transfer. Membranes were then blocked in 5% non-fat dry milk in 1X PBS/0.1% Tween-20 (PBS-T, Amresco) and incubated with primary antibodies overnight at 4°C at optimized dilutions in 5% milk/PBS-T. After washing in PBS-T, membranes were incubated with HRP-conjugated secondary antibodies for 1 h at room temperature at 1:10,000 in 5% milk/PBS-T. Imaging was performed with BioRad ClarityMax ECL substrate or Femto reagent (ThermoFisher Scientific) on a ChemiDoc MP Imaging system (BioRad). The following antibodies were used: MED6 (Santa Cruz sc-390474; 1:500), MED8 (Santa Cruz sc-365960; 1:500), CRSP70 (MED26) (Santa Cruz sc-166614; 1:500), MED1 (Cell Signaling 51613; 1:1,000), MED23 (Novus NB200-339; 1:500), MED24 (Novus NB100-74599; 1:500), CDK8 (Cell Signaling 4101S; 1:250), MED12 (Cell Signaling 4529; 1:1,000), GAPDH (Fitzgerald 10R-G109a; 1:10,000), Myosin Heavy Chain (MF20; DSHB, AB_2147781; 1:1,000), Goat Anti-mouse IgG HRP (Jackson ImmunoResearch 115–035–003; 1:10,000) and Goat Anti-rabbit IgG HRP (Jackson ImmunoResearch 111–035–144; 1:10,000). We adhere to the highest level of rigor using antibodies for western blotting and test each individual antibody reagent prior to the experiment. Each antibody in the study was tested using a range of protein concentrations of each different type of sample. Therefore, the variability in some of the western blots in this study likely reflects differences in the proliferation/differentiation status of the samples and the heterogeneity of tissues from specific experiments.

### 2.9 RNA-seq and snRNA-seq analysis of experimental samples and publicly available datasets

Total RNA was isolated from cultured C2C12 myoblasts and myotubes using Tri-Reagent according to the manufacturer’s instructions (Sigma Aldrich). RNA quality was assessed using a 2,100 Bioanalyzer System (Agilent), and samples with RNA Integrity Number (RIN) greater than 8.0 were sequenced. Illumina RNA-Seq was performed by the Genomics Services Laboratory (GSL) at Nationwide Children’s Hospital (Columbus, OH). Quality assessment of the RNA-Seq data was performed using FastQC (v0.12.0). Quality filtered reads were aligned to the mouse reference genome GRCm38 (mm10) using HISAT2 (v2.2.1). Read counts were obtained from featureCounts in the Subread package (v2.0.6) and z-scores for each gene were calculated using average TPM values and reported as heatmaps.

RNA-seq data were obtained from public repositories. RNA-Seq data from muscle stem cells pre- and post-injury were collected from GSE189073 (Dong et al., 2022) and RNA-Seq reads for mouse muscle aging model were collected from GSE139204 (Ham et al., 2020; Ham et al., 2022; Kaiser et al., 2022). FASTQ files were obtained using the fastq-dump function in the sra-tools package (v3.0.8). Trimgalore! (v0.6.2) was used to prepare the data by trimming adapters. FASTQC (v0.12.0) was used to confirm read quality. HISAT2 (v2.2.1) was used to map the reads to the GRCm38 (mm10) genome. FeatureCounts in the Subread package (v2.0.6) was used to generate raw read counts of individual genes. Z-scores for each gene were calculated using average TPM values and reported as heatmaps.

Single nuclei RNA-sequencing (snRNA-seq) data were obtained from public repositories. snRNA-seq data from mouse developmental hindlimb muscle were collected from GSE211543 (Dos Santos et al., 2023), and snRNA-seq data from wild type (WT) and DMD model mouse tibialis anterior (TA) muscle were obtained from GSE156498 (Chemello et al., 2020). For GSE156498, data were analyzed as previously described (Dos Santos et al., 2023). Briefly, nuclei with fewer than 200 and greater than 4,000 genes and/or with greater than 15,000 reads were removed. Data for each sample were log-transformed and the top 2000 most variable genes were identified independently. Data were then integrated using canonical correlation analysis (CCA) to identify anchor genes. Integrated data were then scaled and linear dimensionality reduction was performed using Principal Component Analysis (PCA). Cells were clustered using the first 15 principal components and a resolution of 0.6. Non-linear dimensionality reduction was performed by Uniform Manifold Approximation and Projection for Dimension Reduction (UMAP). Cluster cell types were manually annotated using marker genes (Chemello et al., 2020). Dot plots were generated from scaled RNA data using the Seurat DotPlot function and ggplot2 (v3.4.4). All analyses were performed with Seurat (v3.1.5) in R (v4.1.1) (Satija et al., 2015; Butler et al., 2018; Stuart et al., 2019; Hao et al., 2021). For GSE211543, nuclei with fewer than 400 and greater than 25,000 reads or with reads mapping to mitochondrial genes at greater than 20% were removed from the analysis. Each sample was then log-transformed and the top 2000 most variable genes were identified independently. All samples were integrated using reciprocal Principal Component Analysis (RPCA) with 20 neighbors used to pick anchors (k = 20). Integrated data were then scaled and linear dimensionality reduction was performed using PCA. Cells were clustered using the SLM algorithm and the first 30 principal components with a resolution of 0.8. Non-linear dimensionality reduction was performed by UMAP. Cluster cell types were manually annotated using marker genes (Dos Santos et al., 2023). Dot plots were generated from scaled RNA data using the Seurat DotPlot function and ggplot2 (v3.4.4). All analyses were performed with Seurat (v4.1.0) in R (v4.1.1) (Satija et al., 2015; Butler et al., 2018; Stuart et al., 2019; Hao et al., 2021). The analysis code used in this study are available on GitHub at https://github.com/dwkolonay/SkMuscMediator.

### 2.10 Statistical analysis

Graphpad Prism was used to graph RT-qPCR data and perform statistical analysis; either one-way ANOVA with Tukey’s multiple comparisons test or Welch’s *t*-test. RT-qPCR data are reported as mean ± standard error of mean (SEM). Western blot quantification was performed using the BioRad ImageLab software. Protein of interest band intensity values were normalized to the corresponding GAPDH loading control band intensity from the same membrane. Normalized band intensities are reported as mean ± standard deviation (SD). Asterisks indicate statistical significance of minimally *p* < 0.05.

## 3 Results

### 3.1 Dynamic gene expression of Mediator complex subunits during developmental myogenesis

To lay the groundwork for investigating Mediator components using the C2C12 cell model of muscle differentiation, we confirmed expression of muscle differentiation-related genes in this system by RNA-seq and RT-qPCR. Myoblast differentiation is a highly controlled process that requires precise transcriptional coordination in a temporal manner (Hernandez-Hernandez et al., 2017; Zammit, 2017). In C2C12 cells, differentiation occurs over a period of several days (Andres and Walsh, 1996), which can be monitored by quantifying myogenic markers. For example, Myogenic differentiation 1 (*Myod1*) is a master transcription factor (TF) for the initial determination of skeletal muscle fate (Tapscott, 2005), and expression of *Myod1* declines during differentiation (Supplementary Figures S1A, B). Myogenin (*Myog*), is a TF that drives terminal muscle cell differentiation (Hernandez-Hernandez et al., 2017; Zammit, 2017), and expression of *Myog* significantly increases early during C2C12 differentiation, but declines in mature myotubes (Supplementary Figures S1A-S1B). Myomaker (*Tmem8c*) is required for myoblast fusion (Millay et al., 2013), and its expression significantly increases during differentiation (Supplementary Figure S1A,B). Myosin heavy chain genes are also differentially expressed during myotube formation and maturation (Soukup and Jirmanova, 2000). Previous studies have shown that myosin heavy chain mRNA isoforms are expressed in distinct temporal patterns during C2C12 myoblast differentiation with expression of embryonic myosin heavy chain (*Myh3*) and neonatal myosin heavy chain (*Myh8*) preceding expression of myosin heavy chain 2X (MyHC-2X; *Myh1*), MyHC-2A (*Myh2*), and MyHC-2B (*Myh4*), which are found predominantly, yet not exclusively, in different fast-twitch muscle fibers (type 2X, 2A, 2B) (Brown et al., 2012). We observed a similar pattern of expression: a robust increase in *Myh3* expression as early as day 1 of differentiation and lagging expression of *Myh1*, *Myh2*, and *Myh4* which steadily increased at later time points during C2C12 myoblast differentiation (Supplementary Figure S1A,B). Molecular markers of differentiation are indicative of formation of myotubes, visible by light microscopy (Supplementary Figure S1C). We also detected similar trends in myogenic markers during primary muscle cell differentiation and observed myotube formation by light microscopy and immunofluorescence staining (Supplementary Figure S2A, B).

**FIGURE 1:**
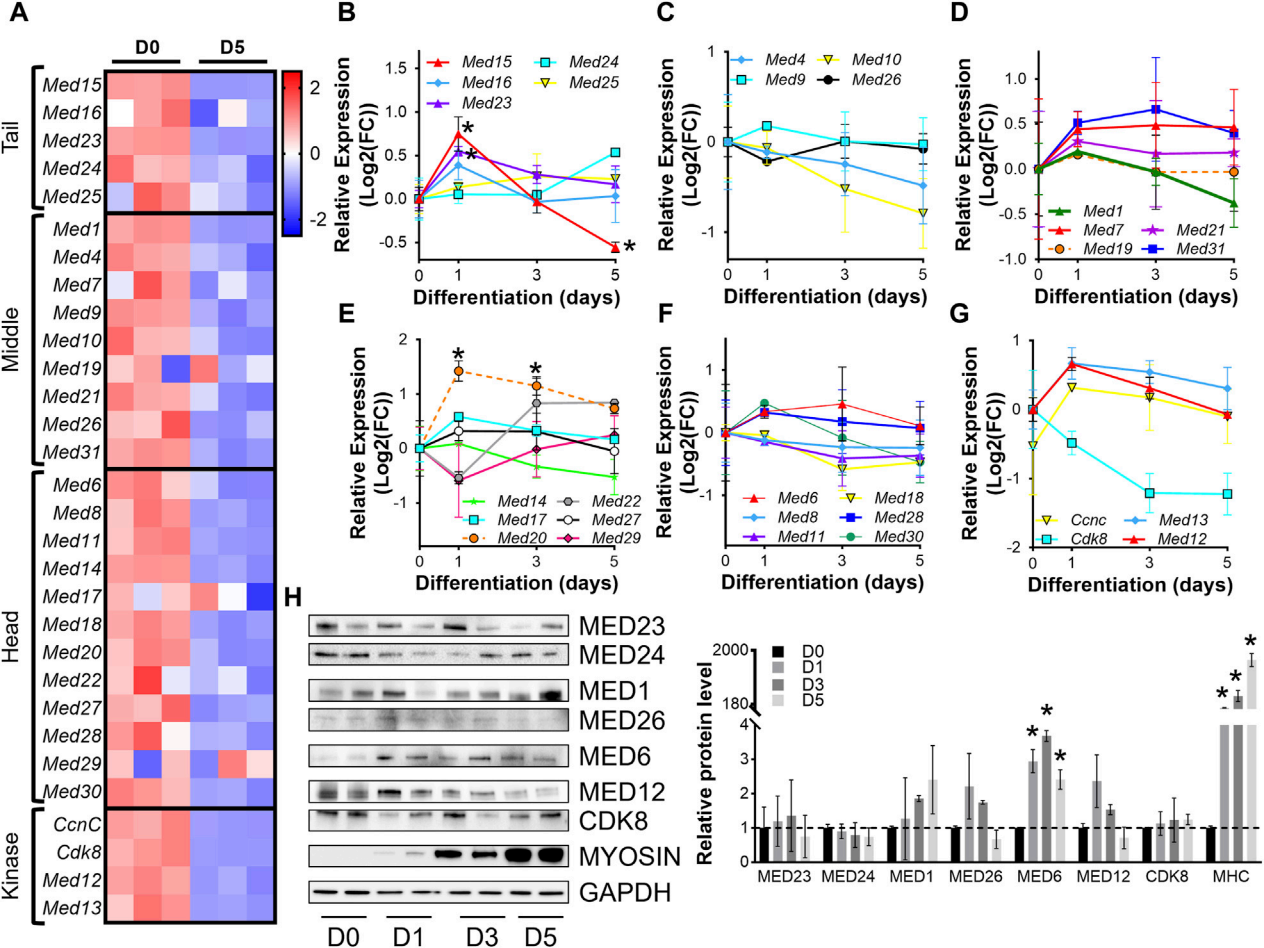
Mediator subunit expression is decreased in differentiated C2C12 myotubes *in vitro*. **(A)** Heat map of z-score–transformed expression of Mediator genes from RNA-seq of C2C12 myoblasts (D0) and myotubes (D5) after 5 days of differentiation. **(B)** RT-qPCR of Tail, **(C,D)** Middle, **(E,F)** Head, and **(G)** Kinase subunits in C2C12 myoblasts (D0) and myotubes after 1, 3, and 5 days of differentiation (D1, D3, D5). **(H)** Protein levels and relative quantification of representative subunits in C2C12 cells before and after differentiation. N = 3/group, *p < 0.05 compared to D0 by one-way ANOVA with Tukey’s multiple comparisons test.

For initial insight into the regulation of the Mediator complex during skeletal muscle differentiation *in vitro*, we analyzed RNA-sequencing (RNA-seq) from C2C12 myoblasts (day 0, D0) and C2C12 myotubes after 5 days (D5) of differentiation. Expression of nearly all Mediator complex subunits were decreased in myotubes (D5) compared to myoblasts (D0) (Figure 1A, Supplementary Table S1). To investigate the temporal regulation of Mediator subunits throughout the process of muscle cell differentiation, we performed RT-qPCR on myoblasts (D0) and during C2C12 myoblast differentiation (D1, D3, D5) (Figures 1B–G, Supplementary Figure S1B). Expression of MED15 and MED16 within the Tail submodule of the Mediator complex significantly increased at the onset of differentiation (D1) but tended to decrease throughout the later stages of differentiation (D3, D5) (Figure 1B). We observed the same pattern of expression for *Med1*, *Med9*, *Med21*, and *Med31* within the Middle submodule (Figures 1C,D); for *Med6*, *Med17*, *Med20*, *Med27*, *Med28*, and *Med30* within the Head submodule (Figures 1E,F); and *Med12*, *Med13*, and *Cdk8* within the Kinase submodule (Figure 1G). Expression of *Med7* (Middle, Figure 1D) remained elevated during differentiation. In contrast to general upregulation of gene expression at the onset of differentiation, expression of *Med4* and *Med10* within the Middle submodule was decreased at D1 and continued to decline throughout differentiation (Figure 1C). Similarly, expression of *Med8*, *Med11*, *Med18*, within the Head submodule (Figure 1F), and *Cdk8* within the Kinase submodule (Figure 1G) also decreased at the onset of differentiation. We also measured protein levels of representative Mediator subunits during myoblast differentiation (Figure 1H). Within the Tail, Middle, and Kinase submodules, MED23, MED24, MED1, MED26, MED12, and CDK8 were not significantly changed during differentiation, indicated by increased abundance of sarcomeric myosin protein levels (Figure 1A). Within the Head submodule we detected significantly higher levels of MED6 during differentiation compared to undifferentiated myoblasts (D0). We detected a significant increase in *Med23* expression but no changes in protein levels of MED23, and we did not detect significant changes in *Med6* expression but observed a significant increase in MED6 protein levels (Figures 1B,F,H). These results reflect the intricacies of differential regulatory steps from transcription to translation including mRNA and protein stability. Unique patterns of Mediator subunit expression throughout muscle cell differentiation may indicate specific temporal functions of different Mediator subunits during muscle differentiation.

**FIGURE 2:**
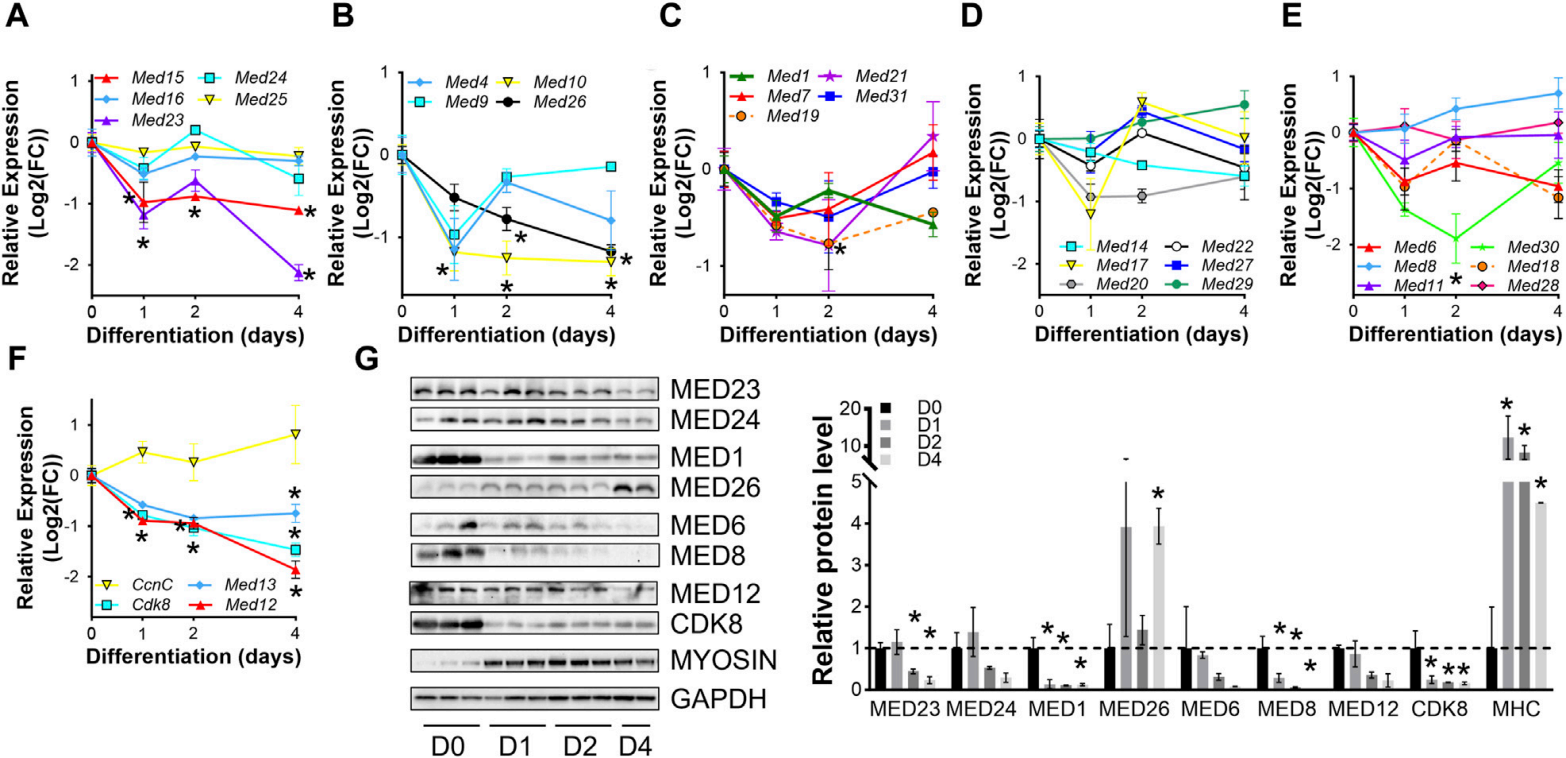
Mediator subunits are temporally regulated during primary mouse myoblast differentiation *in vitro*. **(A)** RT-qPCR of Tail, **(B,C)** Middle, **(D,E)** Head, and **(F)** Kinase subunits in primary myoblasts (D0) and myotubes after 1, 2, and 4 days of differentiation (D1, D2, D4). **(G)** Protein levels and relative quantification of representative subunits in primary muscle cells before and after differentiation. N = 3/group, *p < 0.05 compared to D0 by one-way ANOVA with Tukey’s multiple comparisons test.

To confirm dynamic mediator subunit expression during muscle differentiation in C2C12 cells, we investigated the regulation of Mediator subunits during differentiation of primary mouse myoblasts by RT-qPCR (Figures 2A–G, Supplementary Figure S2). Many Mediator subunits followed a similar expression pattern observed during C2C12 myoblast differentiation: decreased expression after 4 days of differentiation (D4) compared to undifferentiated myoblasts (D0). *Med15* and *Med16* within the Tail submodule (Figure 2A); *Med1*, *Med10*, *Med19*, and *Med26* within the Middle submodule (Figures 2B,C); *Med6*, *Med14*, *Med18*, and *Med20* within the Head submodule (Figures 2D,E); and *Med12*, *Med13* and *Cdk8* within the Kinase submodule (Figure 2F) all followed this expression pattern. In contrast to C2C12 cells, at the very early stage of differentiation, i.e., day 1 (D1), most of these Mediator subunits were drastically downregulated (Figures 2A–F). We attribute the differences in temporal Mediator subunit expression between primary and immortalized C2C12 myoblasts to the inherent differences in myogenic properties of the cells. Specifically, primary myoblasts more readily and quickly differentiate into myotubes compared to C2C12 myoblasts (Grabowska et al., 2011). Additionally, differentiation efficiency is much greater for primary myoblasts than C2C12 myoblasts, ultimately resulting in heterogenetic variability within C2C12 samples (Grabowska et al., 2011). Protein levels of Mediator subunits were also temporally regulated during differentiation of primary myoblasts (Figure 2F). In contrast to our observations in C2C12 cells, MED23 within the Tail submodule, MED1 within the Middle submodule, MED8 within the Head submodule, and CDK8 within the kinase submodule were significantly lower during differentiation of the primary myoblasts. Interestingly, MED26 was more abundant in primary myotubes compared to myoblasts. We detected significantly decreased mRNA expression and protein levels of MED23, MED1, and CDK8, but mRNA expression and protein levels of MED26 and MED8 were oppositely regulated, reflecting the intricacies of differential regulatory steps from transcription to translation in primary mouse myoblasts. Collectively, these data demonstrate that Mediator subunits are temporally regulated throughout muscle cell differentiation.

### 3.2 Mediator complex subunits are highly expressed during embryonic muscle development

**FIGURE 3:**
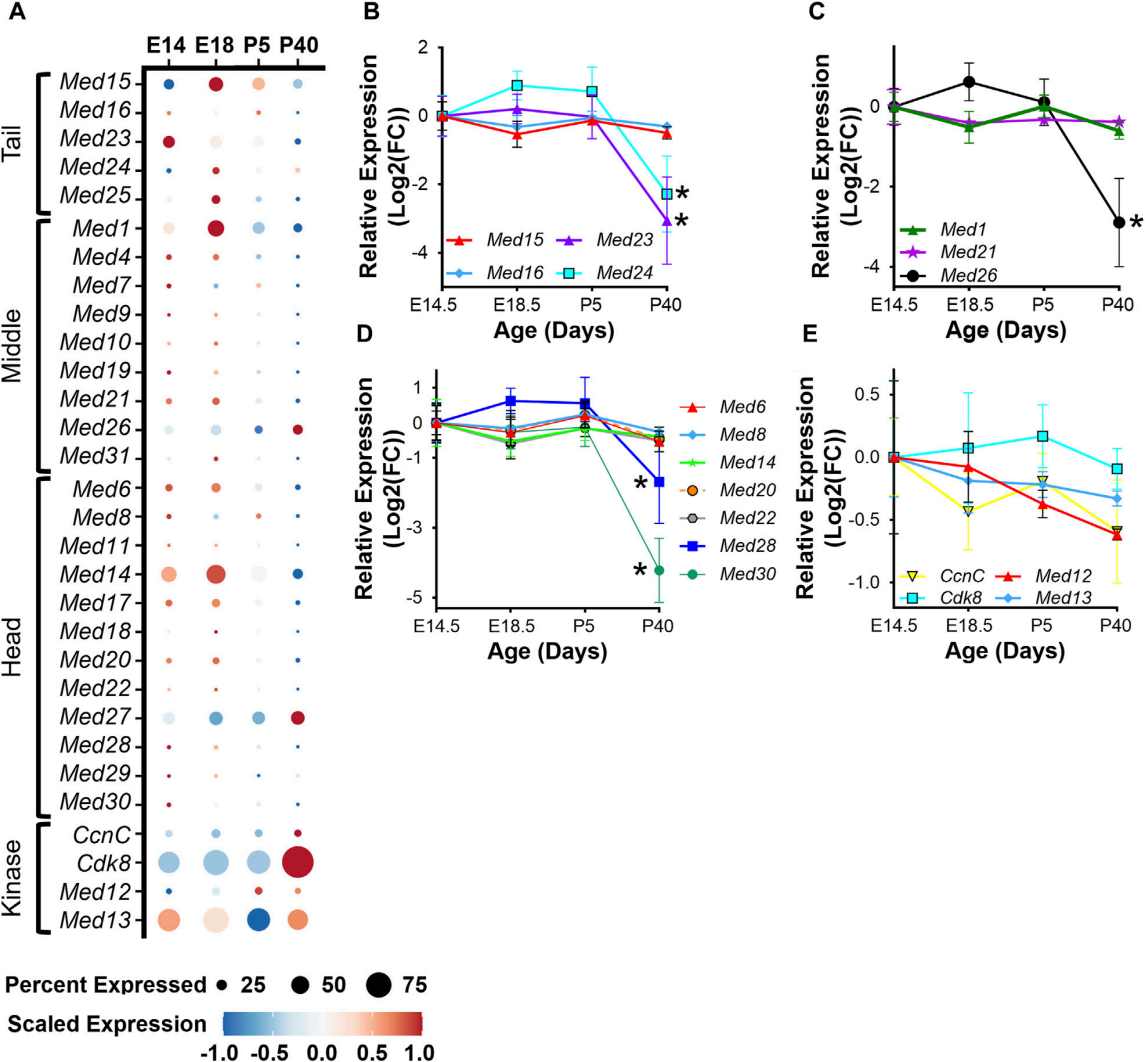
Mediator subunits are highly expressed during muscle development *in vivo*. **(A)** Dot plot of Mediator gene expression from snRNA-seq analysis of pooled mouse whole hindlimb muscle nuclei at embryonic days 14 and 18 and at 5 and 40 days old. **(B)** RT-qPCR of Tail, **(C)** Middle, **(D)** Head, and **(E)** Kinase subunits in mouse muscle (tongue) at embryonic days 14.5 and 18.5 and at postnatal days P5 and P40. N = 3/group, *p < 0.05 compared to E14.5 by one-way ANOVA with Tukey’s multiple comparisons test.

To determine the regulation of Mediator subunits during skeletal muscle development *in vivo*, we analyzed publicly available snRNA-seq data (GSE211543) from mouse hindlimb muscle collected at embryonic day 14 and 18 (E14, E18) and from five- and forty-day-old mice (P5, P40) (Dos Santos et al., 2023). Similar to what we observed during myoblast differentiation *in vitro*, expression of several Mediator subunits increased during hindlimb development from E14 to E18, but then decreased after completion of embryonic myogenesis at P5 and P40. This is most evident for *Med15*, *Med24*, and *Med25* within the Tail; *Med1*, *Med10*, *Med21*, and *Med31* within the Middle; and *Med14*, *Med18*, *Med20*, and *Med22* within the Head submodule (Figure 3A). Interestingly, the expression of Kinase submodule components is not regulated in the same manner, but instead is increased in hindlimb muscle nuclei at P40 (Figure 3A). We also observed decreased expression of MRFs and embryonic and neonatal myosin heavy chain (*Myh3* and *Myh8*), and increased expression of *Myh1*, *Myh2*, *Myh4*, and *Tmem8c* during hindlimb muscle development (Supplementary Figure S3A). To determine if Mediator subunit expression patterns were uniquely regulated in a cell-specific manner, we grouped snRNA-seq Mediator subunit expression by cell type. In general, most Mediator subunit expression patterns within the MuSC and myoblast populations (Supplementary Figure S3B) are similar to the patterns observed in the combined analysis of hindlimb muscle nuclei in Figure 3A. In contrast to MuSCs and myoblasts, expression of most Mediator subunits within the hindlimb myonuclei population is much less dynamic during hindlimb development (Supplementary Figure S3B). This analysis revealed additional complexity of Mediator regulation and shed light onto the cell specificity of Mediator subunit expression dynamics within muscle cell types.

To complement the snRNA-seq analysis of Mediator subunits in developing hindlimb muscle and to explore transcriptional diversity among different muscle types (Terry et al., 2018), we performed RT-qPCR on tongue muscle collected from mice at E14.5, E18.5, P5, and P40. As in hindlimb muscles, expression of some Mediator subunits was decreased in adult tongue muscle (P40) compared to embryonic and post-natal developmental stages (Figures 3B–E). Most notably, expression of *Med23* and *Med24* within the Tail (Figure 3B); *Med26* within the Middle (Figure 3C); and *Med28* and *Med30* within the Head submodules (Figure 3D) significantly decreased in adult tongue muscle. Expression of *Med12* and *CcnC* also trended to decrease in adult tongue muscle (Figure 3E). Taken together, these data are consistent with the hypothesis that increased expression of Mediator subunits may drive muscle cell proliferation in early developmental stages, but lower expression of Mediator subunits may be sufficient to maintain transcriptional control in terminally differentiated myotubes and myofibers.

### 3.3 Mediator complex subunits are temporally regulated during muscle regeneration

**FIGURE 4:**
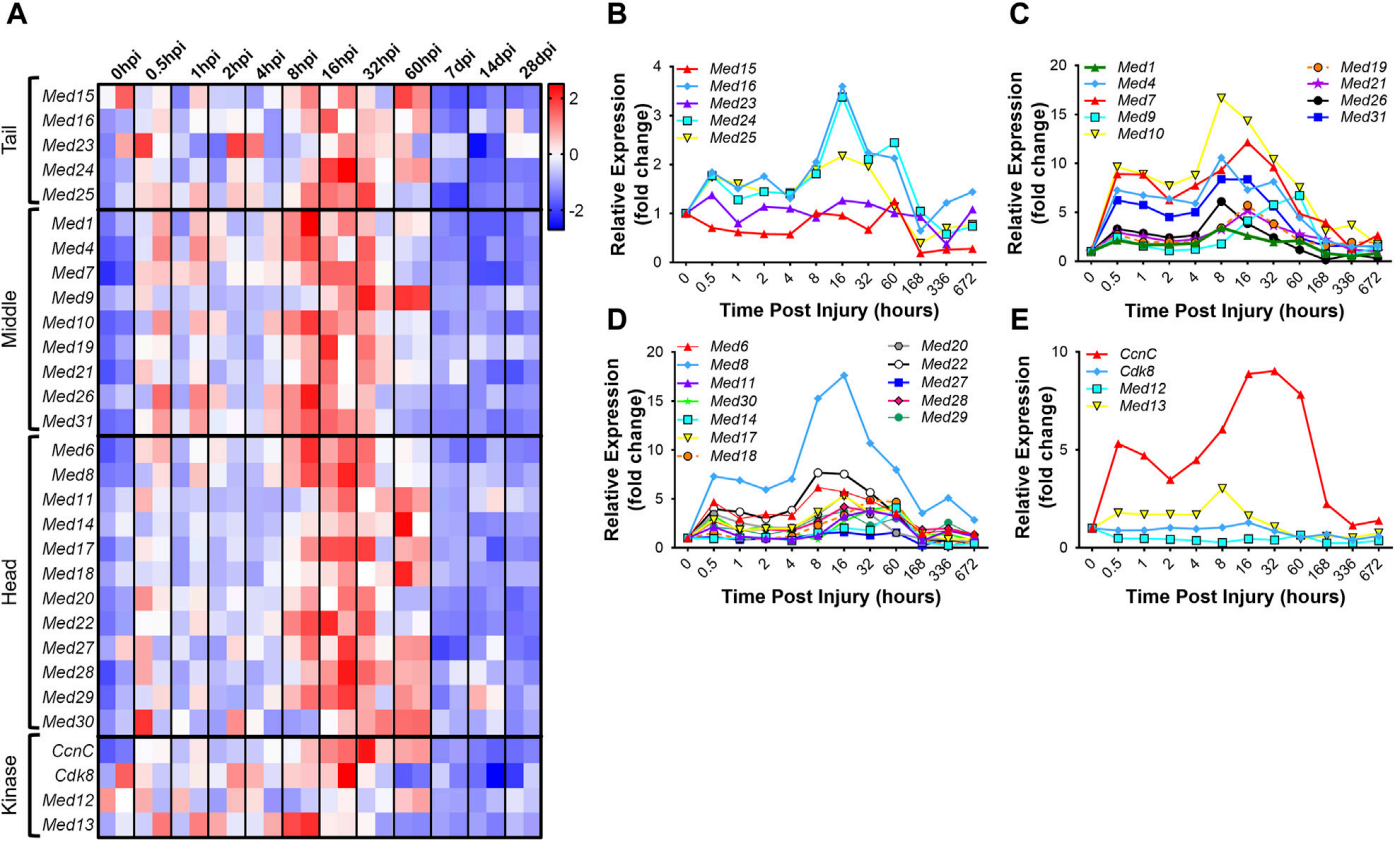
Mediator subunits are temporally regulated in muscle satellite cells after injury *in vivo*. **(A)** Heat map of z-score–transformed expression of Mediator genes from RNA-seq of mouse hindlimb muscles 0–60 h post injury (hpi) and through 28 days post injury (dpi). **(B)** Expression of Mediator genes from RNA-seq of mouse hindlimb muscles 0–672 h post injury (hpi) in the Tail, **(C)** Middle, **(D)** Head and **(E)** Kinase submodules. RNA-seq data are from fixed-sorted satellite cells from N = 2 mice per time point.

Analogous to development, regenerative myogenesis requires myoblast expansion, differentiation, and fusion. Quiescent muscle satellite cells (MuSCs) are activated upon injury, proliferate, differentiate, and then ultimately, fuse with each other to form newly regenerated muscle fiber syncytia (Zammit et al., 2006). To investigate the hypothesis that the Mediator complex is upregulated during muscle cell proliferation *in vivo,* we analyzed publicly available RNA-seq data from an extensive time-course dataset specifically capturing MuSC transcriptomes during early, intermediate and late regeneration time points post-acute injury with barium chloride (GSE189073) (Dong et al., 2022). *In vivo* fixed and sorted MuSCs from mouse hindlimbs were sequenced after muscle injury over the course of 28 days. For most Mediator complex subunits, we observed a bi-phasic increase in expression during muscle regeneration (Figures 4A–E, Supplementary Table S2). The first increase in expression occurred acutely after injury, between 0.5 h post injury (hpi) and 2hpi. The second, more drastic and widespread expression increase occurred between 8hpi and 60hpi. Seven days post injury (dpi), expression of all Mediator subunits returned to levels equal to, or lower than, levels in MuSCs from uninjured muscle (Figures 4A–E). As reported, we also observed temporal regulation of myogenic gene expression. *Myod1* expression increased quickly after injury in activated MuSCs while *Myog* and *Tmem8c* expression increased in the later stages of myotube formation post injury (Supplementary Figure S4A–D). Other MRFs had more pronounced phases of expression throughout the MuSC-mediated injury response (Supplementary Figure S4D), while expression of myosin heavy chain genes was tightly regulated in a temporal manner after injury (Supplementary Figures S4A–C).

**FIGURE 5:**
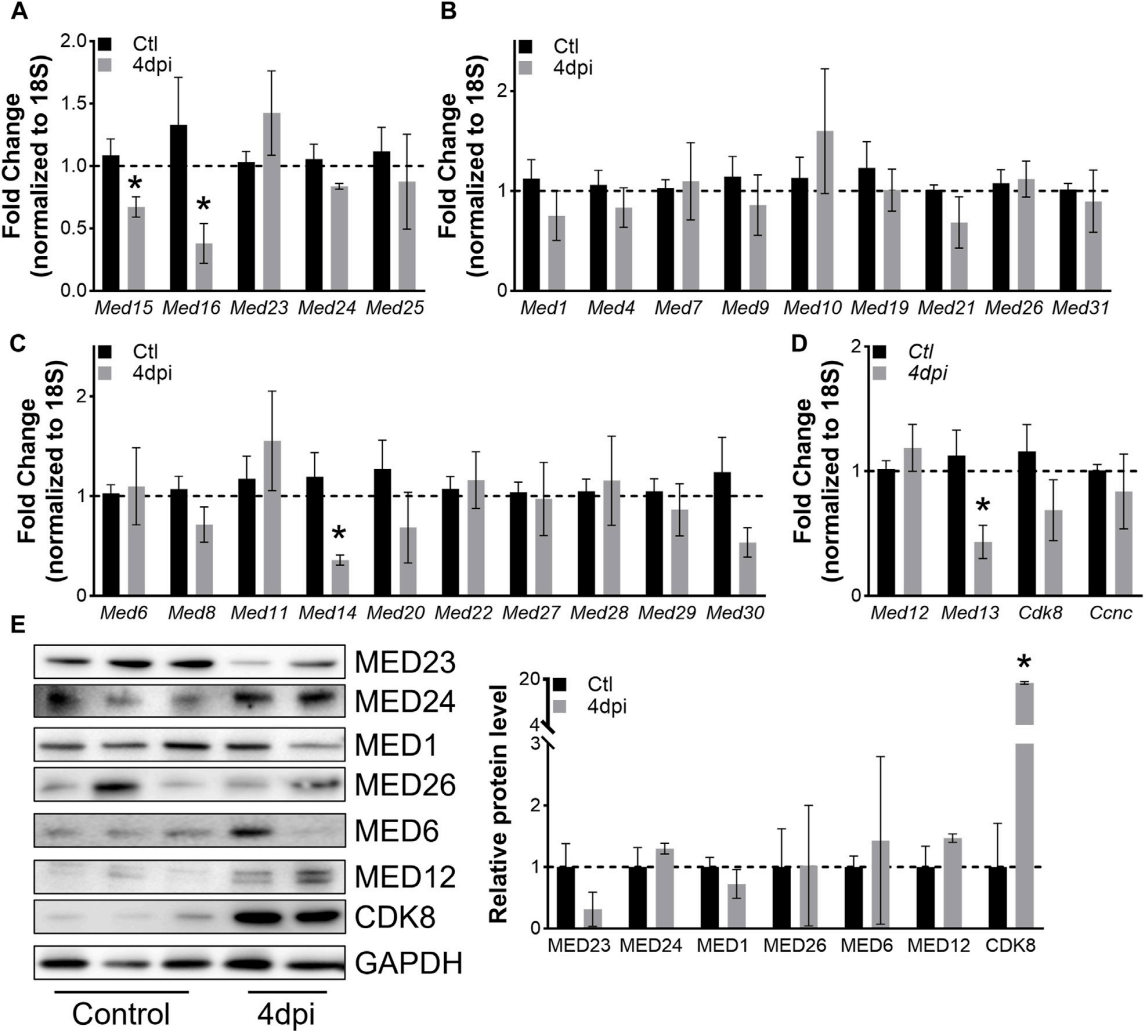
Mediator subunits are differentially regulated in muscles after injury *in vivo*. **(A)** RT-qPCR of Tail, **(B)** Middle, **(C)** Head, and **(D)** Kinase subunits in mouse TA muscles 4 days after injury. **(E)** Protein levels and relative quantification of representative subunits in TA muscles 4 days after injury. N = 4–10/group, *p < 0.05 compared to Control by Welch’s *t*-test.

To explore how levels of Mediator complex subunits change within whole muscles after skeletal muscle injury, we collected TA muscles at 4dpi and performed RT-qPCR. Of note, our analysis used saline-injected contra-lateral TA muscles as controls. Hence, the gene expression signature of our controls contains that of satellite cells, which transitioned out of quiescence into G_alert_ (Rodgers et al., 2014). However, the small fraction of satellite cell-derived RNA is unlikely to impact Mediator gene expression analysis as the RT-qPCR assay lacks that level of sensitivity. Interestingly, we found that expression of only a few subunits were significantly affected in whole muscle after injury. Expression of *Med15* and *Med16* within the Mediator Tail was significantly decreased at 4dpi, while no changes in expression of Mediator subunits within the Middle submodule were detected (Figures 5A,B). Additionally, expression of only *Med14* and *Med13* within the Head and Kinase submodule, respectively, was significantly decreased in TA muscles at 4dpi (Figures 5C,D). We also detected increased expression of muscle differentiation-related genes *Tmem8c* and *Myh3*, and decreased expression of *Myh1* and *Myh2* in TA muscles at 4dpi (Supplementary Figure S5A). We measured protein levels of representative Mediator subunits and observed trending decreased MED23 (Tail submodule) levels in TA muscles at 4dpi (Figure 5E). MED24 (Tail submodule), MED1 and MED6 (Head submodule), and MED12 (Kinase submodule) levels did not significantly change, but we detected a significant increase in CDK8 protein in TA muscles at 4dpi (Figure 5E). These *in vivo* data provide further evidence that expression of Mediator subunits is highly dynamic during skeletal muscle regeneration, which may indicate that they have unique roles in regulating muscle stem cell fate.

### 3.4 Mediator complex subunits are not significantly altered in aging muscle

**FIGURE 6:**
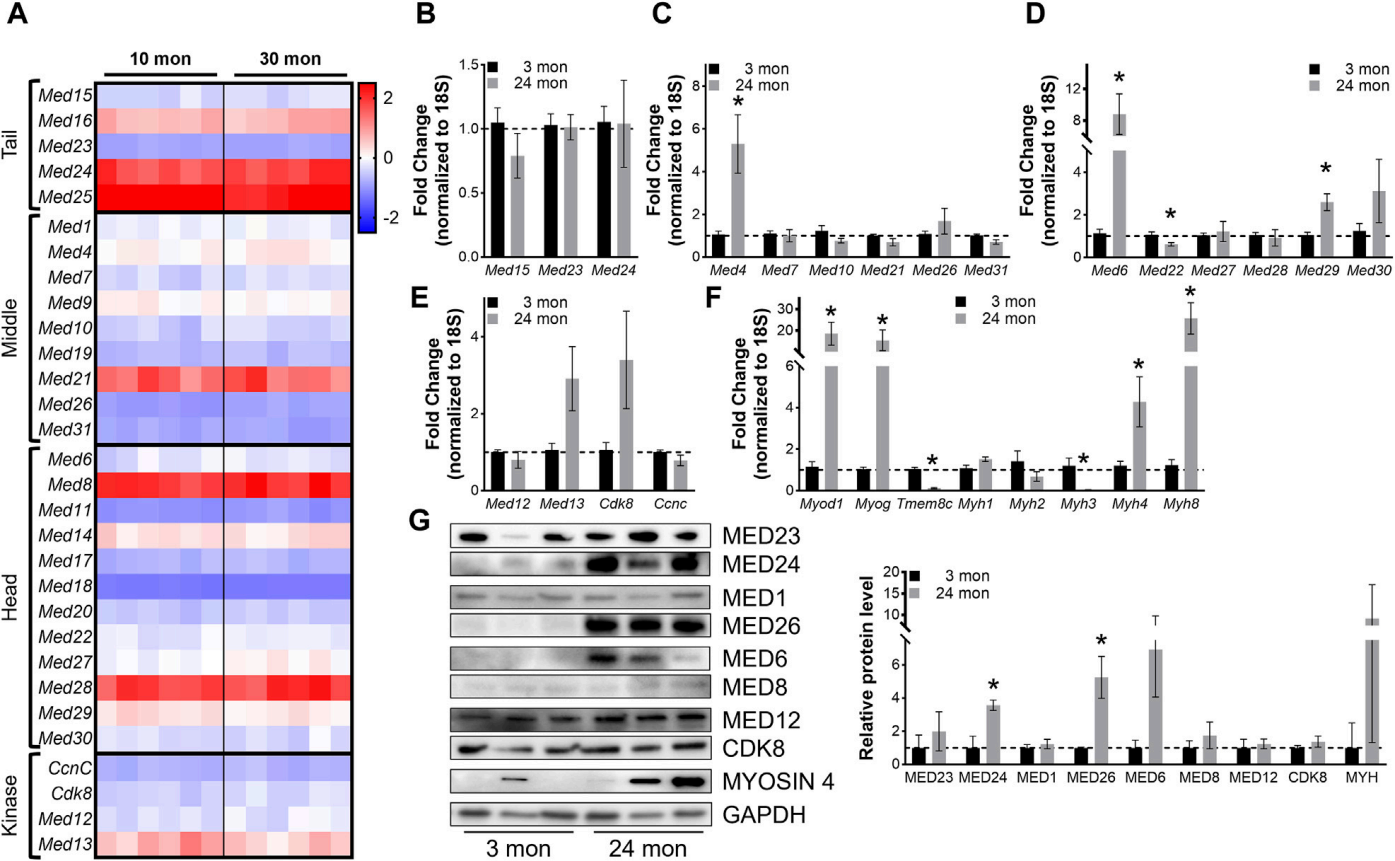
Most Mediator subunits are not altered in aging muscles. **(A)** Heat map of z-score–transformed expression of Mediator genes from RNA-seq of 10- and 30-months-old wild type (Wt) TA muscles. **(B)** RT-qPCR of Tail, **(C)** Middle, **(D)** Head, and **(E)** Kinase subunits of Mediator, and **(F)** myogenic genes in TA muscles from 3- and 24-months-old Wt mice. **(G)** Protein levels and relative quantification of representative subunits in TA muscles from 3- and 24- months-old Wt mice. N = 3/group, *p < 0.05 compared to 3 months by Welch’s *t*-test.

Skeletal muscle atrophy is a hallmark of aging and is characterized by muscle weakness and decreased metabolic flexibility and is also associated with gene expression changes (Distefano and Goodpaster, 2018; Naruse et al., 2023; Shavlakadze et al., 2023). To determine how Mediator complex subunit expression is altered in aging muscles and could contribute to the muscle aging process we analyzed RNA-seq data from 10- and 30-months-old male mouse muscles. Raw sequencing data were used for this analysis (GSE139204) (Ham et al., 2020; Ham et al., 2022; Kaiser et al., 2022). Surprisingly, the expression of most Mediator complex subunits was unaffected by aging in TA muscles (Figure 6A, Supplementary Table S3) as well as in gastrocnemius muscles (Supplementary Figure S6A, Supplementary Table S4), soleus muscles (Supplementary Figure S6B, Supplementary Table S5), and in triceps muscles (Supplementary Figure S6C, Supplementary Table S6). Interestingly, myogenic markers were differentially expressed in aged muscle, in a muscle-specific manner. *Myod1* expression was slightly increased in 30-months-old TA, gastrocnemius, and soleus muscles (Supplementary Figures S6D–F), but not in triceps (Supplementary Figure S6G), while *Myog* expression was increased in all 30-months-old muscles (Supplementary Table S7). Neither *Pax7* nor *Pax3* expression were altered in aged muscles. *Myf6* expression was increased in 30-months-old TA, gastrocnemius, and triceps muscles, but *Myf5* was decreased in aged gastrocnemius muscle. *Myh1* expression was increased, and *Myh4* expression was decreased in aged soleus muscle (Supplementary Figure S6). These findings are in line with previous reports of the negative regulation of skeletal muscle size, which decreases with aging (Shavlakadze et al., 2023). Overall, the general increase in markers of muscle cell differentiation may reflect a disturbed balance of satellite cell quiescence, activation, and differentiation in aged skeletal muscle (Chen et al., 2020; Kurland et al., 2023).

To determine whether expression of Mediator subunits is significantly altered in aging muscle compared to young adult muscle, we performed RT-qPCR on TA muscles collected from 3- and 24-months-old male mice. No differences in Mediator Tail subunits were detected between young and old TAs (Figure 6B), and solely, expression of *Med4* was significantly increased in older TAs within the Mediator Middle submodule (Figure 6C). Within the Tail submodule *Med6* and *Med29* expression was significantly increased and *Med22* was significantly decreased in TAs from older mice (Figure 6D), but no subunits of the Kinase submodule were significantly altered (Figure 6E). We quantified myogenic markers, and analogous to RNAseq data, we observed significantly increased expression of *Myod1* and *Myog* in TAs from 24-months-old mice. Expression of *Myh4* and *Myh8* was also increased, while expression of *Tmem8c* and *Myh3* was decreased (Figure 6F). We also measured protein levels of representative Mediator subunits in TAs from 3- and 24-months-old mice (Figure 6G). MED24 within the Tail submodule, MED26 within the Middle submodule, and MED6 within the Head submodule were significantly increased in old TA muscles (Figure 6G). These *in vivo* data suggest that in general, expression of Mediator subunits is not drastically affected in skeletal muscle during aging, which could be due to the relatively low regenerative state of aging muscles (Munoz-Canoves et al., 2020).

### 3.5 Mediator complex subunits are dysregulated in mouse models of Duchenne Muscular Dystrophy

**FIGURE 7:**
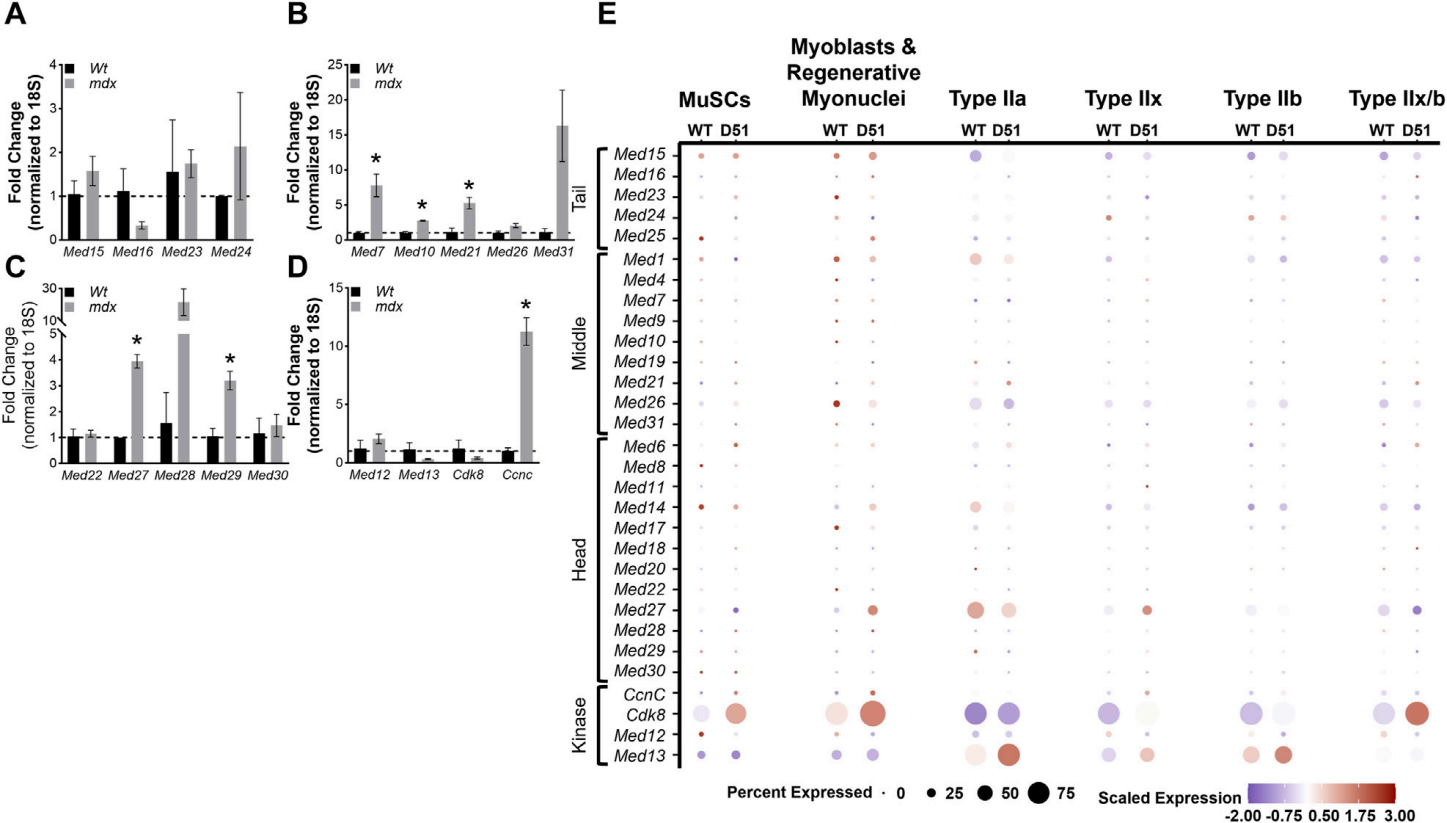
Mediator subunits are differentially expressed in muscles from two mouse models of Duchenne muscular dystrophy (DMD). **(A)** RT-qPCR of Tail, **(B)** Middle, **(C)** Head, and **(D)** Kinase subunits in TA muscles from 8–10 week old WT and *mdx* mice. **(E)** Dot plot of Mediator genes from snRNA-seq of 4-week-old Wt and D51 TA muscle nuclei grouped by cell type. N = 3/group, *p < 0.05 compared to WT by Welch’s *t*-test.

Duchenne Muscular Dystrophy (DMD) is a fatal muscle disorder caused by mutations in the dystrophin gene (*DMD*) that results in cycles of muscle fiber degeneration and regeneration (Darras et al., 1993). The dystrophin-deficient *mdx* mouse model mimics DMD pathology and is widely used to investigate DMD (Bulfield et al., 1984; Partridge, 2013). To determine how Mediator complex subunits are altered during chronic degeneration and regeneration cycles of dystrophic muscle, we performed RT-qPCR on TA muscles from Wt and *mdx* mice. We did not detect any significant differences in expression of Mediator Tail subunits in *mdx* compared to Wt TA muscle (Figure 7A). Many of the subunits of the Mediator Middle submodule were increased in *mdx* TA muscles including *Med7*, *Med10*, and *Med21* (Figure 7B). We also detected significantly increased expression of *Med27* and *Med29* within the Head submodule, and *CcnC* within the Kinase submodule (Figures 7C,D). Expression levels of myogenic genes were also increased, including *Myod1*, *Tmem8c*, *Myh3*, and *Myh8* reflective of ongoing myogenesis (Supplementary Figure S7).

Deletion of exon 51 (D51) within the *Dmd* gene in mice also recapitulates aspects of the disease pathology observed in DMD (Chemello et al., 2020). To determine how Mediator complex subunits are altered during the chronic degeneration and regeneration cycles of dystrophic muscle, we analyzed single nuclei RNA-sequencing (snRNA-seq) data from 4-week-old TA muscles isolated from wild type (WT) mice and mice lacking *Dmd* exon 51 (D51). Processed sequencing data was used for this analysis (GSE156498) (Chemello et al., 2020). We observed many different patterns of expression between Mediator subunits within different muscle cell types. Interestingly, *Med16* and *Med23* (Tail submodule); *Med19*, *Med21*, *Med26*, and *Med31* (Middle submodule); *Med6*, *Med11*, *Med17*, *Med18*, and *Med28* (Head submodule); and *CcnC* and *Cdk8* (Kinase submodule) displayed increased expression in D51 MuSCs (Figure 7E), which reflects the increased expression of Mediator subunits we observed in MuSCs during regeneration after muscle injury (Figure 4A). Expression of *Med16*, *Med25*, *Med21*, *Med14*, *Med27*, *Med28*, *CcnC*, and *Cdk8* also increased in D51 myoblasts and regenerative myonuclei (Figure 7E). However, only *Med15*, *Med24*, *Med21*, *Med6*, *Med17*, and *Med13* increased in D51 Type IIa nuclei (Figure 7E). *Med15*, *Med1*, *Med4*, *Med7*, *Med19*, *Med6*, *Med11*, *Med14*, *Med27*, *Med28*, *Med29*, *CcnC*, *Cdk8*, and *Med13* increased in D51 Type IIx nuclei, while *Med15*, *Med16*, *Med7*, *Med21*, *Med6*, *Med14*, *Med22*, *Med27*, *CcnC*, *Cdk8*, and *Med13* increased in D51 Type IIb nuclei (Figure 7E). Lastly, *Med15*, *Med16*, *Med25*, *Med21*, *Med26*, *Med6*, *Med8*, *Med18*, *Med22* and *Cdk8* increased in D51 Type IIx/b nuclei (Figure 7E). Taken together, this analysis provides a glimpse of the complex regulation of Mediator subunit expression in myonuclei and serves as a springboard for mechanistic studies into the function of individual Mediator subunits in skeletal muscle.

### 3.6 Discussion

Developmental stages of skeletal muscle are temporally well-defined, and the transcriptional regulation of muscle development by key transcription factors is well established. However, it is not clear how TFs are coordinated to regulate gene expression during skeletal muscle development, regeneration, and disease. Based on our studies and other published evidence, we hypothesize that the Mediator complex is a critical regulator and coordinator of transcription in skeletal muscle. In this study, we investigated the temporal regulation of Mediator subunits during muscle proliferation, differentiation, regeneration, aging, and disease. Our findings demonstrate that expression of mediator subunits is not static. Instead, individual components can be dynamically regulated depending on the physiologic and/or temporal context, which has important implications for the mechanistic understanding of transcriptional regulation of skeletal muscle.

Previous studies using genetic mouse models support a role for the Mediator complex in regulating embryonic stem cell and hematopoietic stem cell fate. One of the major findings of our study is that Mediator subunits are temporally regulated throughout muscle cell differentiation, suggesting that Mediator may contribute to directing muscle stem cell fate. We observed dynamic regulation of Mediator complex subunit expression during myogenesis *in vitro*, both in the C2C12 muscle cell line and in primary mouse muscle cells (Figure 1; Figure 2). We confirmed our *in vitro* findings by demonstrating that Mediator subunits are also dynamically regulated during embryonic muscle development (Figure 3).

Muscle regeneration after injury requires MuSC activation, proliferation, differentiation, and ultimately fusion, as well as stem cell self-renewal to maintain the tissue’s regenerative capacity. In line with our observations during muscle development, Mediator subunit expression is highly dynamic during muscle regeneration (Figure 4; Figure 5). We uncovered unique temporal patterns of Mediator expression in MuSCs just after injury and throughout the early regeneration period, which may indicate that Mediator subunits could uniquely contribute to directing muscle stem cell fate. Widespread acutely upregulated expression of several Mediator subunits in MuSCs within 24 h post injury potentially indicates a role for Mediator in establishing the transcriptional landscape for MuSC amplification. MuSC transcription programs change dramatically and swiftly during the initial phase of injury-induced regeneration to support MuSCs breaking quiescence, activating and initiating rapid proliferation cycles (Dong et al., 2022). The robust transition from a transcriptional program supporting the quiescent MuSC state to activated transient amplifying myoblast state likely requires precise coordination by Mediator complex. The dynamic expression levels of several Mediator subunits may be a critical contributing factor to fine-tuning of Mediator activity for stem cell state transitioning.

Given the dynamic expression patterns of Mediator subunits during muscle development and regeneration, it is not surprising that we observed few changes in Mediator subunit expression in aging muscles compared to younger muscles (Figure 6). These results suggest that the lower regenerative state of aging muscles requires less dynamic expression of Mediator subunits to maintain muscle homeostasis. Consistent with this supposition, we observed vast heterogeneity of Mediator subunit expression in dystrophic muscle nuclei, indicative of chronic muscle degeneration and regeneration cycles (Figure 7).

Here, we systematically uncovered Mediator subunit expression patterns throughout the life cycle of skeletal muscle. We demonstrate that Mediator subunits are temporally regulated throughout muscle cell differentiation, which suggests that each subunit could play an important role in regulating muscle development and/or regeneration. Despite our findings, and despite the importance of Mediator in transcriptional control previously reported in other cell types, only MED1 and MED13 have been investigated in skeletal muscle thus far. *Med1*, a Mediator subunit of the Middle submodule, is required for embryonic development, and it broadly regulates transcription through nuclear receptors, rendering MED1 critical for metabolic gene regulation (Fondell et al., 1996; Ito et al., 2000; Zhu et al., 2000; Chen and Roeder, 2011; Jia et al., 2014). Skeletal muscle-specific deletion of *Med1* (MKO) increases mitochondrial density in quadriceps resulting in a switch towards slow muscle fibers. Consequently, *Med1* MKO mice are resistant to high-fat diet (HFD)-induced obesity and display enhanced insulin sensitivity and improved glucose tolerance (Chen et al., 2010). *Med13*, a subunit of the Kinase submodule, is also required for embryonic development (Miao et al., 2018). In contrast to *Med1* MKO mice, skeletal muscle-specific deletion of *Med13* (-mKO) does not affect muscle histology or function. However, in response to HFD, *Med13*-mKO mice have improved glucose tolerance and are protected from hyperinsulinemia and hepatic steatosis, due in part to increased glucose handling gene expression in skeletal muscle (Amoasii et al., 2016). These studies highlight the importance of individual Mediator subunits for skeletal muscle development and/or physiologic adaptation.

The functions of a few Mediator subunits have been uncovered in other cells and tissues, and interestingly, several subunits contribute to the regulation of metabolism. For example, MED13 in the heart affects cardiac metabolism and systemic metabolism (Grueter et al., 2012; Baskin et al., 2014). MED1 is also an important regulator of cardiac metabolism gene expression, and CCNC regulates cardiac mitochondrial dynamics and lipid metabolism in brown adipose tissue (Spitler et al., 2017; Ponce et al., 2020; Song et al., 2022). Additionally, both MED1 and MED23 have been implicated in regulating liver metabolism (Chu et al., 2014; Jia et al., 2014). Given the high metabolic activity of skeletal muscle, it will be important to tease out the functions of individual Mediator complex subunits in skeletal muscle in future studies (Baskin et al., 2015).

In summary, our studies reveal that Mediator subunits are dynamically and differentially regulated in skeletal muscle under numerous physiologic and temporal contexts. Little is known about each Mediator subunit, and we provide the first comprehensive overview identifying subunits which may hold important implications in skeletal muscle development, regeneration, aging, and disease. Given the reliance of transcriptional regulation to initiate and control these processes within muscle, it is intriguing to speculate that Mediator subunits act to fine-tune Mediator activity within each of these contexts. Our findings provide a segue to initiate in-depth investigations into individual Mediator component functions in skeletal muscle.

## Data Availability

The data presented in the study are deposited in in the Gene Expression Omnibus repository, accession number GSE247438. The datasets analyzed for this study can be found in the Gene Expression Omnibus and include GSE189074, GSE139204, GSE211543, GSE156498.
