# Supplementary material for: Temporal regulation of the Mediator complex during muscle proliferation, differentiation, regeneration, aging, and disease
Source: Front Cell Dev Biol. 2024 Apr 16;12:1331563. doi: 10.3389/fcell.2024.1331563 (PMC11058648; doi:10.3389/fcell.2024.1331563)
Supplement: Supplementary file 8 [file DataSheet1.docx]

Supplementary Material

MED-iating skeletal muscle development and regeneration

Dominic W. Kolonay, Kristina M. Sattler, Corinne Strawser, Jill Rafael-Fortney, Maria M. Mihaylova, Katherine E. Miller, Christoph Lepper*, Kedryn K. Baskin*

*** Correspondence:**

Kedryn K. Baskin: [Kedryn.Baskin@osumc.edu](mailto:Kedryn.Baskin@osumc.edu)

Christoph Lepper: [Christoph.Lepper@osumc.edu](mailto:Christoph.Lepper@osumc.edu)

# Supplementary Tables and Figures


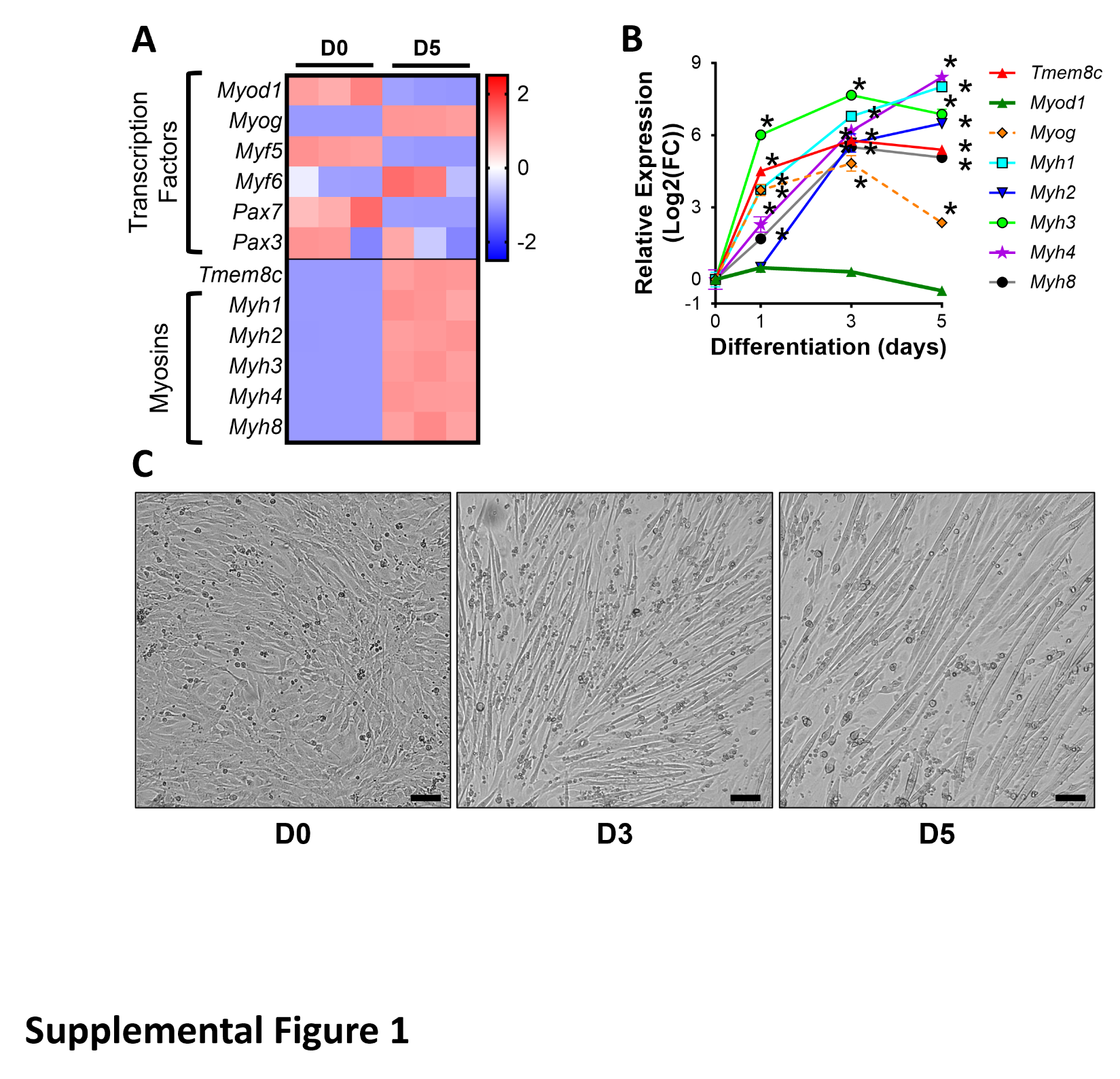


**Supplemental Figure 1.** Myogenic markers are highly expressed during C2C12 differentiation *in vitro*. (A) Heat map of z-score–transformed expression of myogenic marker genes from RNA-seq of C2C12 myoblast (D0) and myotubes (D5) after 5 days of differentiation. (B) qPCR of myogenic marker genes in C2C12 myoblast (D0) and myotubes after 1, 3, and 5 days of differentiation (D1, D3, D5). (C) Representative brightfield live cell images of C2C12 myoblasts (D0) and myotubes (D3, D5) during differentiation. Scale bar 200μm. N=3/group, *p<0.05 compared to D0 by one-way ANOVA with Tukey’s multiple comparisons test.


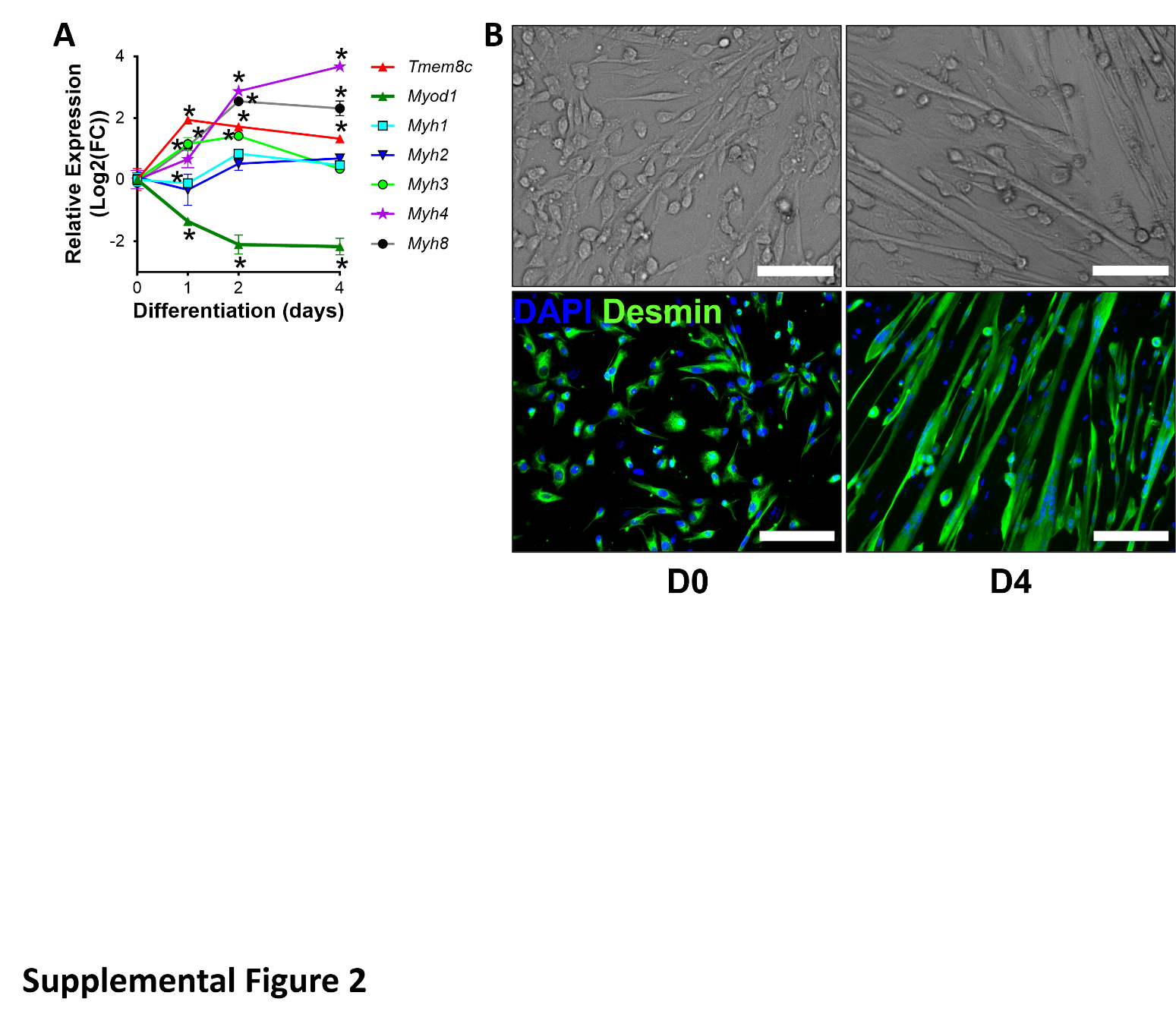


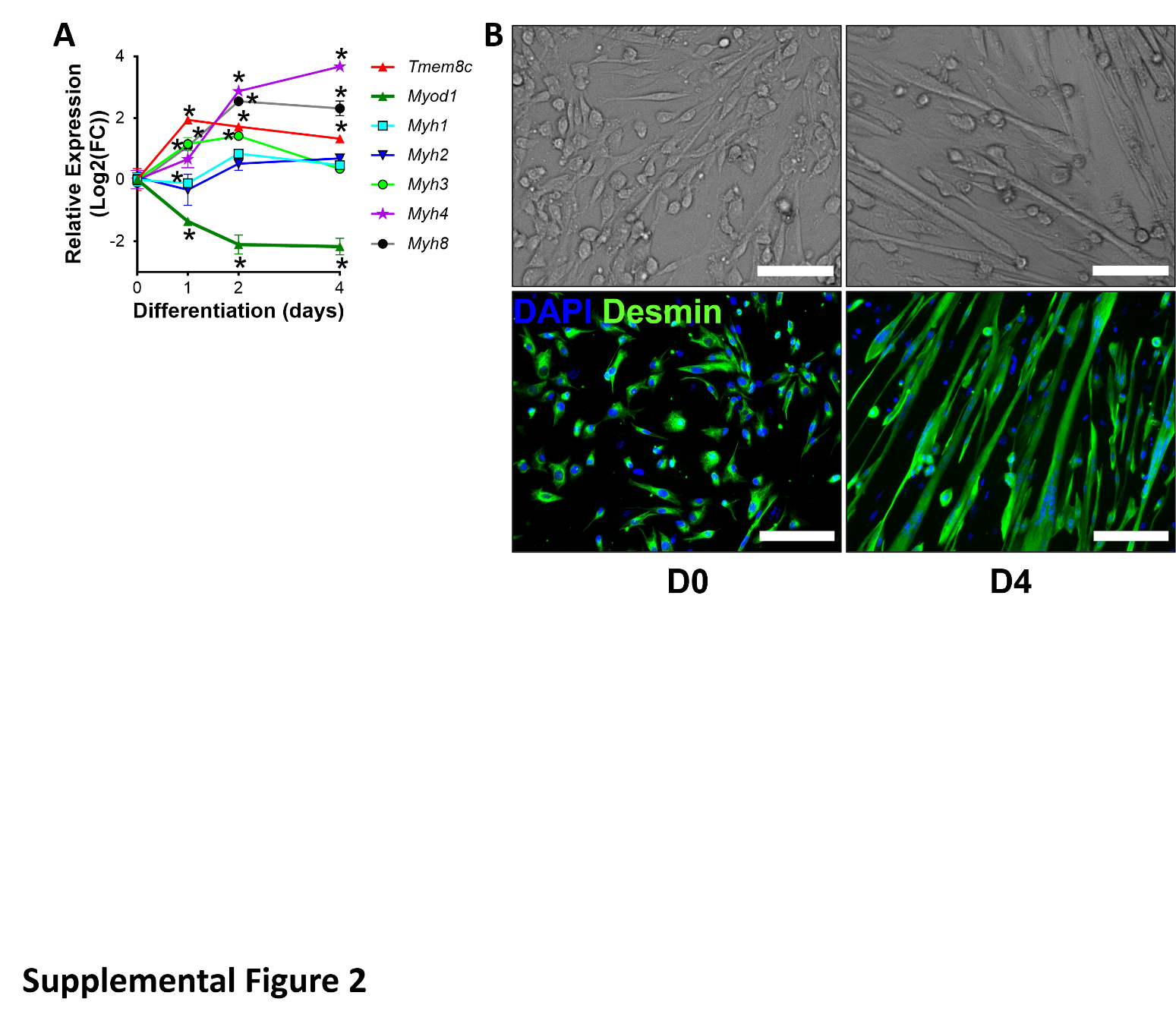


**Supplemental Figure 2.** Myogenic markers are highly expressed during primary mouse myoblast differentiation *in vitro*. (A) qPCR of myogenic marker genes in primary myoblasts (D0) and myotubes after 1, 2, and 4 days of differentiation (D1, D2, D4). (B) Representative brightfield and immunofluorescence images of primary myoblasts (D0) and myotubes (D4) during differentiation. Scale bar 100μm. N=3/group, *p<0.05 compared to D0 by one-way ANOVA with Tukey’s multiple comparisons test.

**
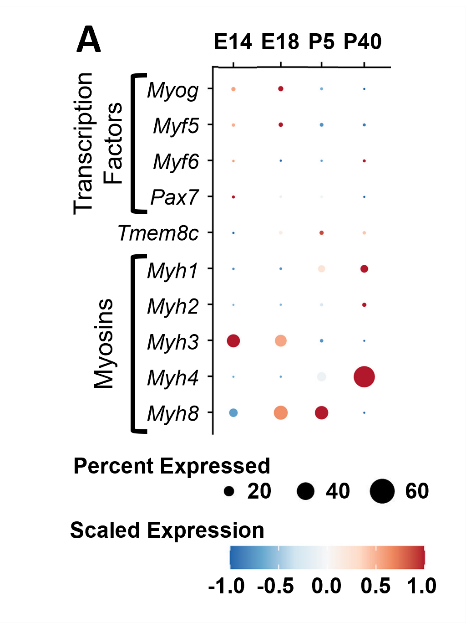

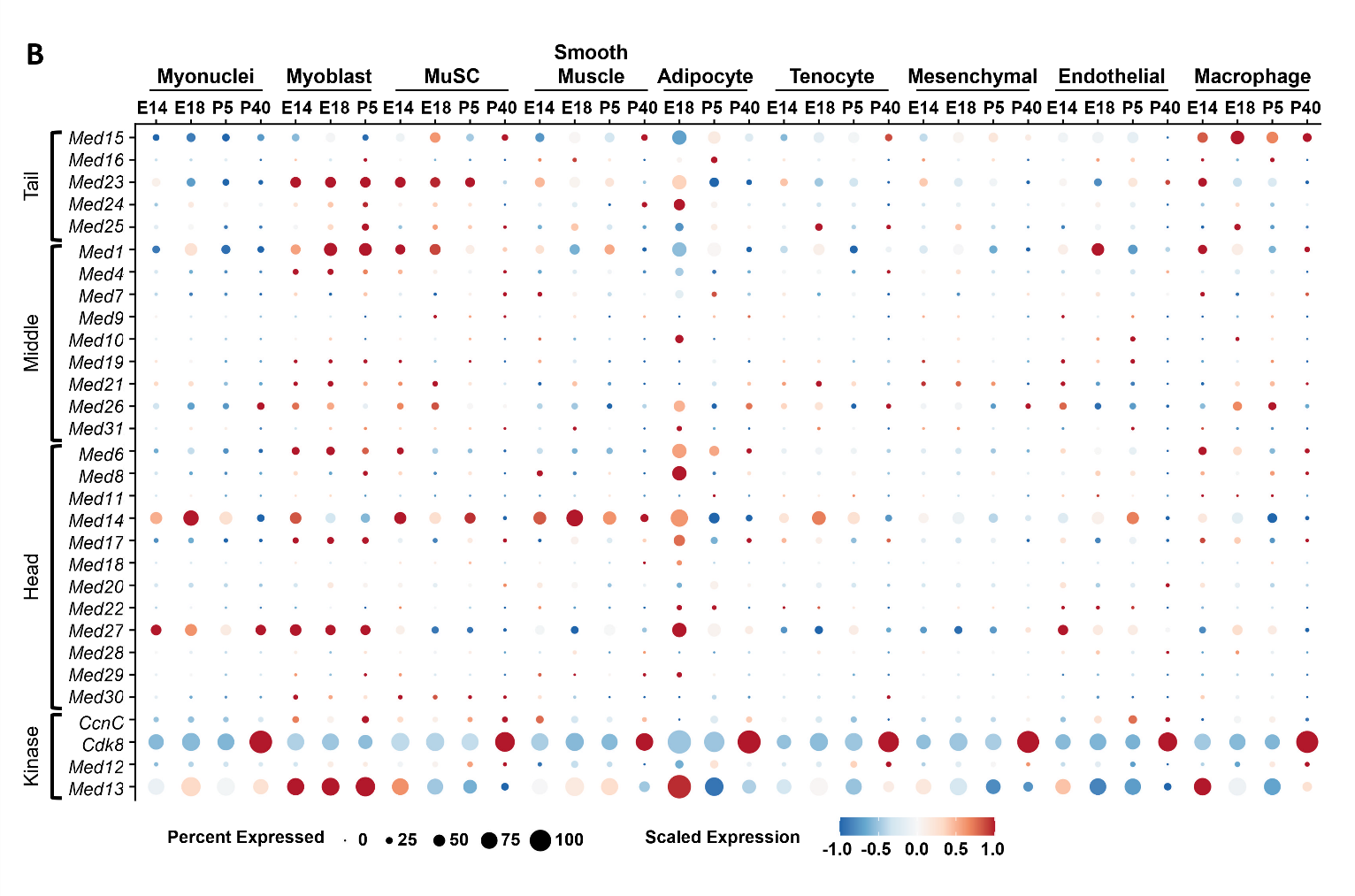
**

**Supplemental Figure 3.** Myogenic markers are highly expressed during muscle development *in vivo*. (A) Dot plot of myogenic marker gene expression from snRNA-seq of mouse whole hindlimb muscle nuclei at embryonic days 14 and 18 and at 5 and 40 days old. (B) Dot plot of Mediator gene expression from snRNA-seq of mouse whole hindlimb muscle nuclei at embryonic days 14 and 18 and at 5 and 40 days old, grouped by cell type.


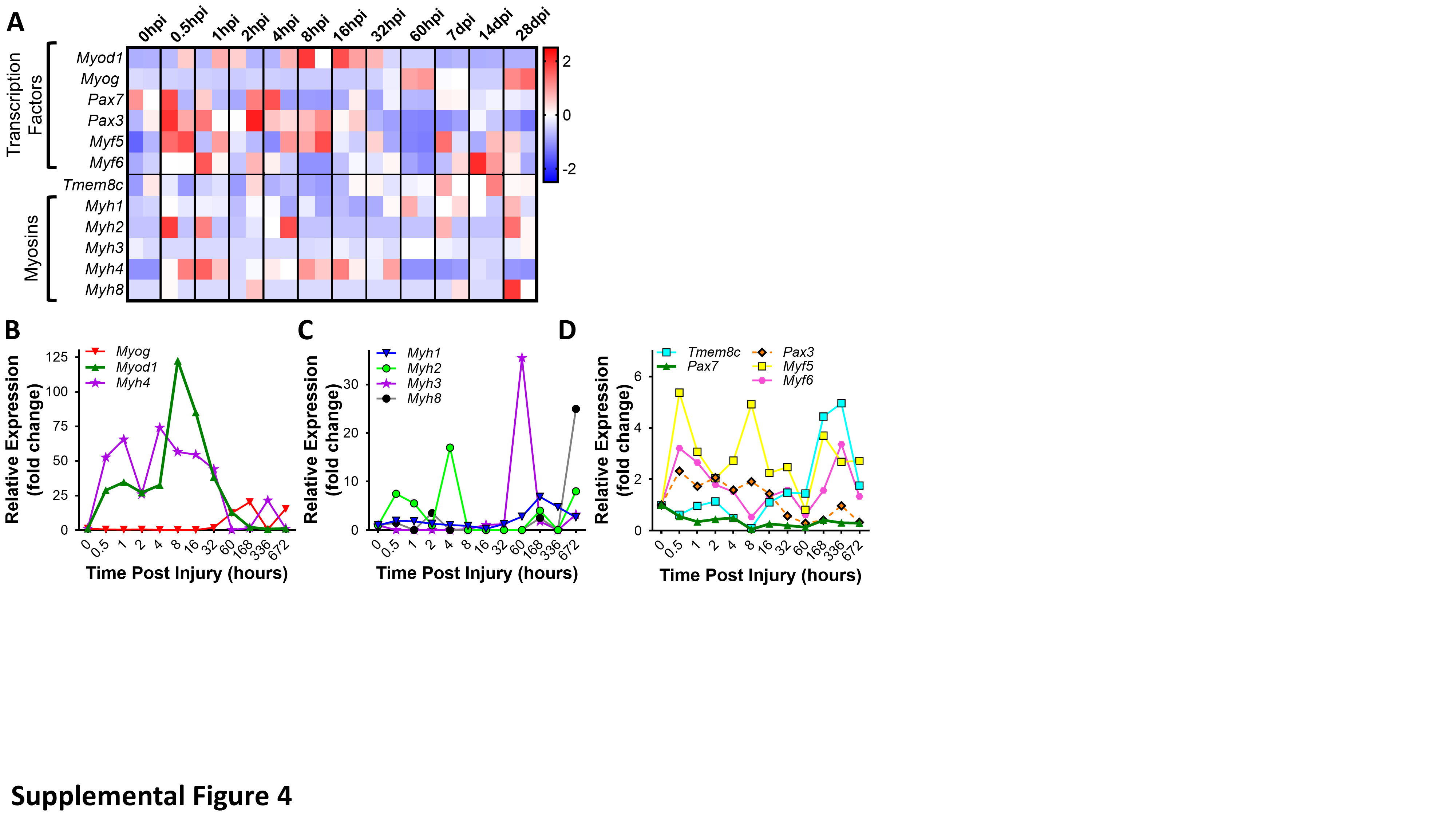


**Supplemental Figure 4.** Myogenic markers are dynamically expressed in muscle satellite cells after injury *in vivo*. (A) Heat map of z-score–transformed, and (B), (C), (D) expression of myogenic genes from RNA-seq of mouse hindlimb muscles 0-672 hours post-injury (hpi). RNA-seq data are from fixed-sorted satellite cells from N=2 mice per time point.


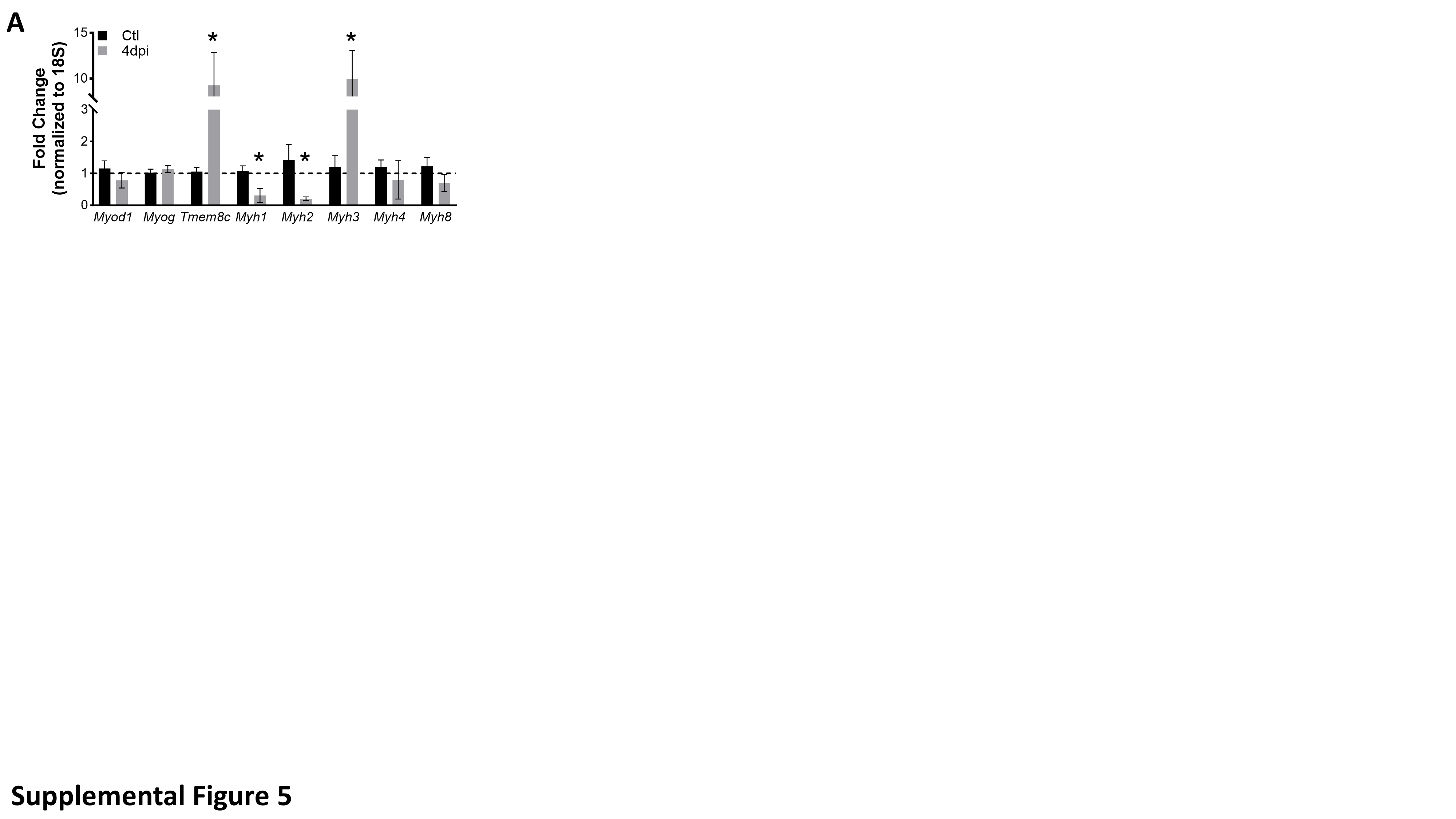


**Supplemental Figure 5.** Myogenic markers are differentially expressed in muscles after injury *in vivo*. (A) RT-qPCR of myogenic genes in mouse TA muscles 4 days post-injury. N=4-10/group, *p<0.05 compared to Control by Welch’s *t* test.


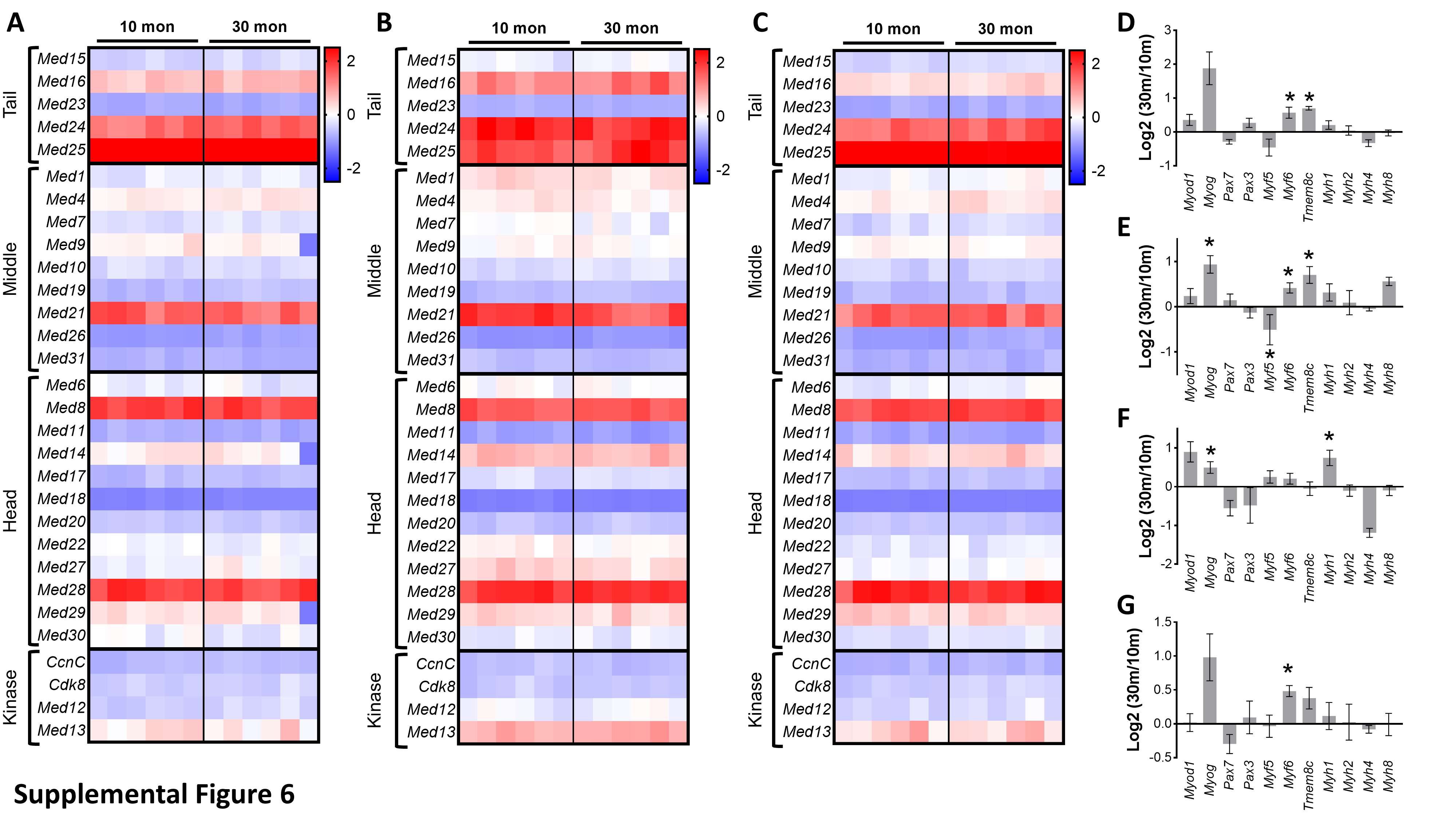


**
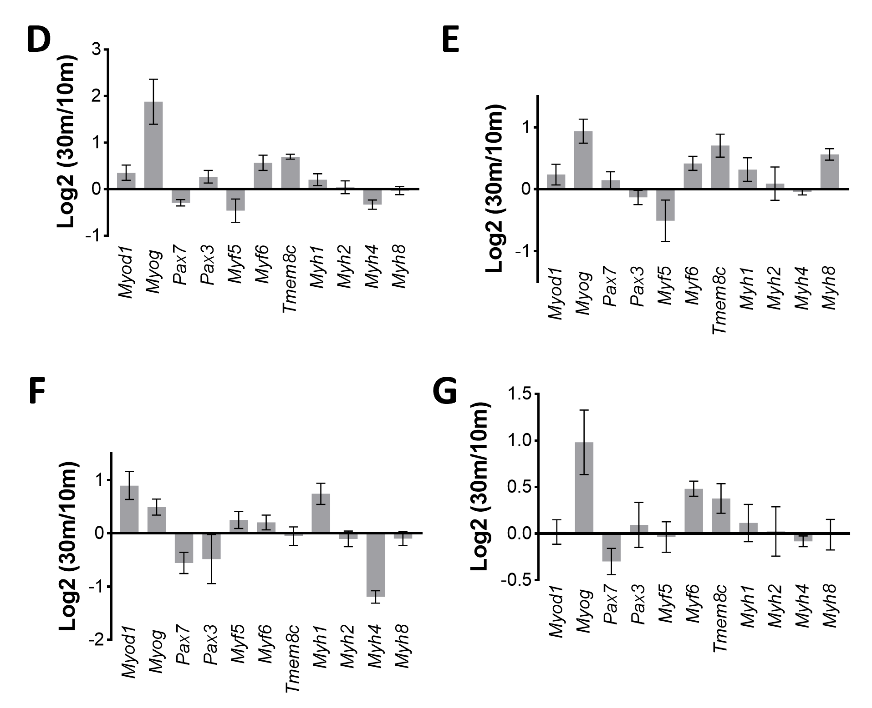
**

**Supplemental Figure 6.** Few Mediator subunits are altered in aging muscles. (A) Heat map of z-score–transformed expression of Mediator genes from RNA-seq of 10- and 30-months-old Wt gastrocnemius muscles. (B) Heat map of z-score–transformed expression of Mediator genes from RNA-seq of 10- and 30-months-old Wt soleus muscles. (C) Heat map of z-score–transformed expression of Mediator genes from RNA-seq of 10- and 30-months-old Wt triceps muscles. (D) Expression of myogenic markers from RNA-seq in TA, (E) gastrocnemius, (F) soleus and (G) triceps muscles. N=5-6/group.


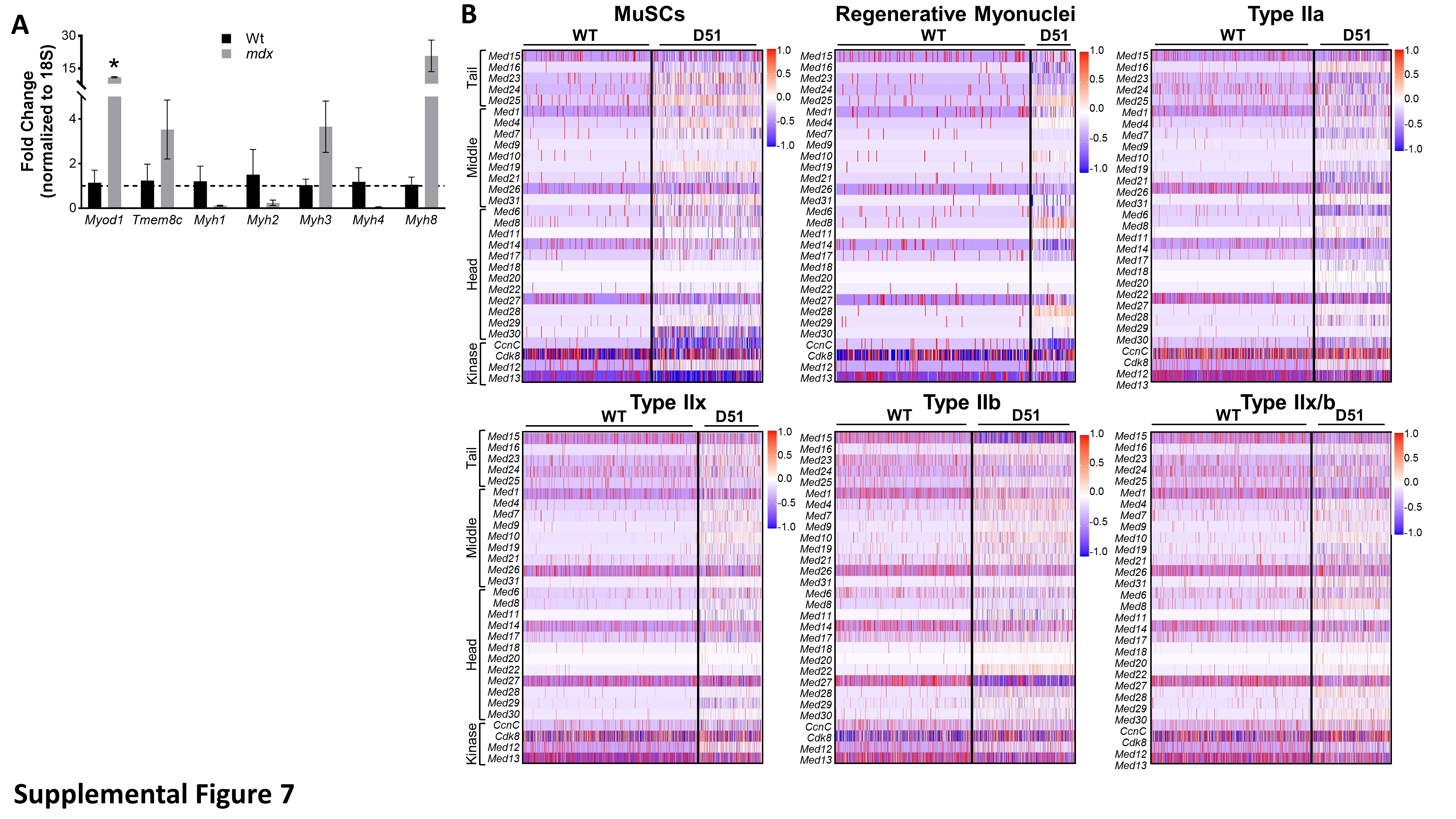


**Supplemental Figure 7.** Mediator subunits are differentially expressed in muscles from a model of Duchenne muscular dystrophy (DMD). (A) RT-qPCR of myogenic genes from 8-10-week-old Wt and *mdx* mouse TA. N=3/group, *p<0.05 compared to Wt by Welch’s *t* test.
